# Integrated analysis of single-cell RNA-seq and bulk RNA-seq reveals *MMP* mediated expression patterns by distinct tumor microenvironment immune profiles in cervical cancer

**DOI:** 10.7150/jca.96429

**Published:** 2024-08-13

**Authors:** He Wang, Xinbo Li, Siyu Zhou, Wendi Guo, Zhao Wang, Linlin Sun, Zhongyi Zhao, Yanyan Han, Sanyuan Zhang, Jieping Lv, Yi Ping, Zhe Wang

**Affiliations:** 1 Second Hospital of Shanxi Medical University, Department of Obstetrics and Gynecology, Taiyuan 030001, China.; 2 First Hospital of Shanxi Medical University, Department of Gynecology, Taiyuan 030001, China.; 3 Department of Gynecology, Jiaocheng County People's Hospital, No. 25 Tianning Street, Jiaocheng County, Lüliang City, Shanxi Province, China.; 4 First Hospital of Shanxi Medical University, Department of Anesthesiology, Taiyuan 030001, China.; 5 Nankai University, The School of Medicine, Tianjin 300071, China.

**Keywords:** Matrix metalloproteinase, Cervical cancer, Tumor microenvironment, Extracellular matrix, Single-cell sequencing, Immunotherapy

## Abstract

**Background:** Few studies have analyzed the effect of *matrix metalloproteinase* (*MMP*) expression patterns on the tumor microenvironment (TME) during development of cervical cancer (CC).

**Methods:** We elucidated the landscape and score of *MMP* expression in CC using single-cell RNA sequencing (scRNA-seq) and RNA sequencing datasets. Further, we aimed the MMPscore to probe the infiltration of immune cells. Further, *MMP* expression was measured by quantitative Real-Time Polymerase Chain Reaction (qRT-PCR).

**Results:** We found *MMPs* were cell-type specific expressed in diverse types of CC cells, regulating the relative pathways of CC progression. Two distinct *MMP* expression patterns that associated infiltrated tumor microenvironment (TME) were identified. We discovered *MMP* expression patterns can predict the stage of tumor, subtype, stromal activity in the TME, genetic variation, and patient outcome. Patients with high MMPscore benefited from significantly better treatment and clinical outcomes.

**Conclusion:** These results indicate high MMPscore in diverse cell types may regulate immune response and improve the survival of patients with CC, which assist in developing more effective immunization strategies.

## Introduction

Cervical cancer (CC) is the most common gynecological malignancy. Risk factors for CC include human papillomavirus (HPV) infection, long-term unhealthy lifestyle maintenance, eating habits, vaginal microenvironment, and regional differences 1-4. Although the use of HPV vaccines has been promoted in recent years, the global incidence of CC remains high. According to the American Cancer Society, the estimated number of new CC cases in the United States reached 14,100, and the estimated number of deaths will reach 4,280 in 2022, compared with 14,480 and 4,290, respectively, in 2021 5. In 2020, the diagnosis rate of CC in Europe was 58.2/100,000, with a mortality rate of 26.0/100,000 6. The citywide CC incidence in Guangzhou, China, displayed an annual increase of 2.1% from 2004 to 2018, with the most substantial increase in rural areas, where average annual percentage change of 6.6% 7. In recent years, the proportion of young patients with CC has increased. The incidence of undiagnosed early-stage squamous cell carcinoma has consistently increased in young women aged < 50 years in Japanese metropolitan areas 8. Invasive and distant metastases in CC are associated with a substantially low five-year survival rate. Thus, developing an index to assess tumor aggressiveness is crucial to evaluating the prognosis of patients with CC and guiding their clinical treatment.

The tumor microenvironment (TME) is the cellular environment in which tumors or tumor stem cells exist, including tumor cells, adipocytes, fibroblasts, lymphocytes, dendritic cells, cancer-associated fibroblasts, and tumor vasculature, and is widely associated with tumorigenesis. These cells interact with circulatory and lymphatic systems to promote tumorigenesis and progression 9-11. Single-cell transcriptomics has revealed heterogeneity in tumor- and tumor-derived endothelial cells in CC 12. Other cancers exhibit heterogeneity in tumor cells and tumor-associated cells. Cancer-associated fibroblasts of different origins contribute to the heterogeneity of tumor cells and exert functional effects on tumors through various mechanisms 13, 14. Tumor-associated neutrophils are associated with the heterogeneity of lung cancer cells 15. In breast cancer, tumor-associated macrophages exhibit significant heterogeneity and comprise anti-tumor M1-like tumor-associated macrophages (TAM) or pro-tumor M2-like TAM 16, 17.

Two critical stages of tumor development are the degradation of the basement membrane and the invasion of tumor cells into the surrounding tissues. Metastasis of cancer cells is a complex multistep process involving changes in intercellular adhesion, degradation of the extracellular matrix (ECM) and basement membrane, detachment of tumor cells in situ, and extensive infiltration of proteolytic enzymes into lymphatics or blood vessels, which ultimately enhance the ability of tumor cells to invade and metastasize. Matrix metalloproteinases (MMPs) are a series of diverse protein enzymes involved in ECM degradation, mainly by degrading collagen IV and laminin. Various MMPs are produced by tumor and tumor-related cells. During tumor development, MMPs enable tumor cells to cross the matrix membrane barrier and increase their invasiveness, migration, and metastasis, thereby promoting tumor progression. MMP-9 secreted by neutrophils, mast cells, and macrophages degrade the main components of the basement membrane to promote tumor invasion 18-20. Fibroblasts and tumor cells can secrete MMP-13, MMP-7, and MMP-14. MMP-13 promotes tumor angiogenesis 21, MMP-7 degrades heparin binding epidermal growth factor and E-cadherin in the basement membrane 22, 23, and MMP-14 degrades CD-44 and electron-cadherin in the basement membrane 24, 25. Together, these MMPs play a vital role in tumor invasion. MMP-10 is highly expressed in squamous cells and promotes the recruitment of infiltrating cells by remodeling the ECM. MMP-10 can also upregulate the expression of MMP-7, MMP-9, and MMP-13, which are critical for tumor progression 26. MMPs are essential for neovascularization, the inflammatory response, and apoptosis, and play a major role in leukocyte infiltration and tissue inflammation. In the TME, MMP-1 and MMP-2 secreted by fibroblasts promote tumor growth 27, 28. MMP-3 secreted by fibroblasts is an essential mediator of tumor angiogenesis and progression 27. Collectively, MMPs play an important role in regulating the mechanisms of tumor metastasis.

However, the difference in the expression of MMPs secreted by heterogeneous tumor cells and tumor-related cells, as well as the relationship between the expression level of MMPs and the occurrence and progression of CC, remains unclear. Furthermore, its predictive effect on the prognosis of CC also remains unexplored. In this study, we used single-cell RNA sequencing (scRNA-seq) datasets, including patients with CC and healthy individuals, to analyze the *MMP* landscape at the single cell level. We discovered that *MMPs* displayed cell-type-specific expression patterns in CC cervix tissues. We analyzed differentially expressed *MMPs* and their association with the infiltration of immune cells. We quantified the *MMP* expression of individual tumor cells by constructing the MMPscore to assess the effect of heterogeneity in* MMP* expression. We believe our study would assist in evaluating whether the MMPscore can effectively predict the prognosis of patients with CC and its significance in guiding the clinical treatment of patients with CC.

## Materials and Methods

### Single-cell sequencing

Single-cell RNA sequencing (scRNA-seq) datasets for CC and benign cervical lesion (BCL) cells were obtained from the Gene Expression Omnibus (GEO) (ID: GSE168652). The data came from one CC patient and one BCL patient, and sequencing was done using the 10x Genomics Chromium platform. The UMAP algorithm was used for data visualization, and an unsupervised clustering algorithm classified the cells. Differentially expressed genes (DEGs) were identified using the FindMarkers function in Seurat to accurately annotate cell types, and we manually curated genetic markers for each cell type. Most markers that distinguished between different cell types were retrieved from the Cell Marker Database (https://www.labome.com/method/Cell-Markers.html), with the following cutoff thresholds: Benjamin-Hochberg's adjusted *p* value < 0.01 and Fold Change > 1.5. The DEGs were analyzed for Gene Ontology (GO) enrichment using the clusterProfiler package, with pathways having an adjusted *p*-value < 0.05 deemed significantly enriched. A gene set enrichment analysis (GSEA) was also conducted to identify enriched gene sets within specific cell clusters. Additionally, scRNA-seq datasets from the ArrayExpress database (accession E-MTAB-11948), including three CC and three BCL samples, were used to explore correlations between *MMP* regulatory factors and key pathways in CC tissue.

### CC dataset origin and pretreatment

In our study, we sourced gene-expression data and clinical annotations from the GEO and the Cancer Genome Atlas (TCGA) database, excluding patients without survival data. Cancer genome map from TCGA database, including 261 CC samples and three BCL samples, we identified co-expressed genes in the two expression modes of MMPcluster-A and MMPcluster-B, constructed Venn diagrams, and analyzed the copy number variation, somatic mutation data, and survival information. With gene expression summary from GEO database (ID: GSE192897), we retrieved RNA-seq data from 16 patients, encompassing 11 patients with CC and 5 with BCL. The clinical information obtained included sex, age, tumor stage, grading, and survival analysis.

### Unsupervised clustering of different *MMP* expression levels

Through copy number variation (CNV) analysis, three differentially expressed *MMPs* were selected between cervical cancer CC and BCL samples. Seven additional *MMPs*, closely linked to CC, were chosen through literature review. Heatmaps, Spearman analyses, and correlation networks confirmed the association of these 10 *MMPs*. Spearman's correlation analysis was conducted to ascertain the relationship between the 10 *MMP* phenotypes and CC, defining two distinct expression patterns by unsupervised clustering: MMPcluster-A and MMPcluster-B. Cluster number and stability were validated using the consensus clustering algorithm, and principal component analysis verified the presence of these two patterns. The ConsensusClusterPlus package was utilized with 1,000 iterations to ensure classification stability 29.

### Enrichment Analysis (GSVA) *MMPs* modifier mode and functional analysis of different phenotypes

The biological process differences between the two *MMP* expression modes were analyzed using GSVA enrichment analysis via the "GSVA" R package. GSVA, a nonparametric and unsupervised method, estimates pathway and biological process activity changes in expression datasets 30. The gene set "c2.cp.kegg.V6.2.Symbols" was downloaded from the MSigDB database for use with GSVA (https://www.gsea-msigdb.org/gsea/msigdb/), with an adjusted *p*-value < 0.05 indicating statistical significance. Functional annotation of *MMP*-related genes was performed using the clusterProfiler R package, applying an FDR cutoff < 0.05.

### Tables Estimation of TME cell infiltration

The single-sample gene-set enrichment analysis (ssGSEA) algorithm was applied to measure the relative abundance of various immune cell infiltrations in the CC TME. A gene set, compiled by Charoentong, labeling distinct TME-infiltrating immune cell types was utilized, encompassing a range of human immune cell subtypes such as activated dendritic cells, CD8+ T cells, natural killer T cells, regulatory T cells, and macrophages 31,32. The relative abundance of each TME-infiltrated cell in each sample was represented by the enrichment fraction calculated by ssGSEA analysis.

### Typing of *MMP* phenotype co-expression gene

Initially, the "limma" R package was used to identify DEGs between CC and BCL tissues. From these DEGs, we selected those with expression patterns specific to MMPcluster-A and MMPcluster-B. Unsupervised clustering then delineated three distinct *MMP* phenotypes, designated as *MMP* gene clusters A-C, aiding in the characterization of unique *MMP* expression profiles and patient categorization for subsequent analyses.

### Generation of* MMP* gene signature

Module scores and the enrichment fraction for *MMP*-related gene expression in single cells were calculated using the AddModuleScores function in the scRNA-Seq database. A scoring system was developed to assess the *MMP* expression pattern in individual cervical cancer patients, allowing quantification of the *MMP* expression profile in single tumors. To establish the MMPscore, the procedure included processing the MMPcluster-A and MMPcluster-B expression patterns, then extracting the overlapping genes. Patients were subsequently grouped using unsupervised clustering for detailed analysis of these overlapping DEGs. The consensus clustering algorithm was used to determine both the number and stability of gene clusters. A univariate Cox regression model was applied to analyze the prognosis associated with each gene. Genes showing significant prognostic impact underwent further analysis. Principal Component Analysis (PCA) was used to create *MMP*-related gene signatures, where the first two principal components served as signature scores. This method emphasized genes with strong positive or negative correlations within the set, while down-weighting contributions from genes didn't track with other set members. Similarly to the Gene Global Index (GGI) method, we defined the MMPscore 33, 34.:



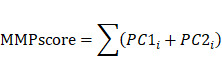



where is the expression of *MMP* genes.

### Relevance of *MMP* gene traits to other relevant biological processes

We identified genes linked to CC biological processes, such as immune checkpoints and antigen processing, using public databases. Further analysis disclosed connections between *MMP*-related gene features and TME immune cells, highlighting associations with relevant biological pathways.

### Genomic and clinical information on immune checkpoint inhibitors

First, by systematically searching the immune checkpoints to block the gene expression spectrum, two immunotherapy cohorts were included: PD-1 and CTLA4, as representatives of combined immunotherapy. Thus, *MMP* expression patterns can predict a patient's response to immune checkpoint blockade therapy.

### Patients and data collection

Patients for the CC study were recruited from eligible women residing in Shanxi province who underwent CC screening. Those diagnosed with non-typical squamous cells of uncertain significance through liquid-based cytology were included, with BCL cells forming the control group. Individuals showing abnormal Pap test results underwent colposcopy and histopathological examination. The study encompassed three CC and three BCL samples. The following participants were excluded: (1) pregnant women, (2) patients with a history of hysterectomy, (3) patients with a history of treatment for cervical and vaginal lesions; (4) patients with other malignant tumors; and (5) patients with blood and digestive system disorders. Ethical approval was granted by the Second Hospital of Shanxi Medical University's Ethics Committee (Approval No.: 2023 YX No. 158), and all participants provided written informed consent. All CC patients received colposcopy, histopathological assessment, and HPV nucleic acid testing and typing. BCL patients underwent similar histopathological and HPV tests. Samples for BCL patients were taken from the cervix during total hysterectomies for uterine fibroids. Age did not significantly differ between the two patient groups (*p* > 0.05).

For CC tissue, a gynecologist with over two years of experience performed colposcopy. To minimize diagnostic deviations, two additional tissue samples (approximately 5 mm each) were collected using forceps. These samples were rinsed with physiological saline and placed in an RNA preservation and tissue fixation solution. Samples for quantitative Real-Time Polymerase Chain Reaction (qRT-PCR) were stored overnight at 4°C and then transferred to a -20°C freezer for long-term storage. Hematoxylin-eosin (HE) stained samples were kept at room temperature.

### Hematoxylin-eosin staining (HE)

Samples were fixed overnight at room temperature or 37°C using a paraformaldehyde fixing solution and dehydrated using a Histo-Tek VP1 dehydrator (Sakura, Japan). Tissue samples were then embedded in paraffin, placed in a freezer until fully set, and sectioned at 3-5 µm using a LEICA RM2235 paraffin microtome (LEICA, Germany). Slice preparation: The TKY-TKB slice baking machine (Taikang, Hubei, China) was used to spread, flatten, and dry the slices. Samples were incubated at 60°C for 2-3 hours, then cooled before dewaxing. Dewaxing and rehydration were performed as follows: xylene (three 10-minute washes), 100% alcohol (two 5-minute washes), 95%, 80%, and 70% alcohol (each for 5 minutes), followed by distilled water and PBS (three 5-minute washes each). Hematoxylin staining (40 seconds) was applied, followed by differentiation with ethanol hydrochloride (1 second), and rinsing with tap water (three to five times). Following staining, samples were dyed with eosin (40 seconds), rinsed with tap water (3-5 times), and dehydrated with 70% alcohol (10 seconds), 80% alcohol (30 seconds to 1 minute), 95% alcohol (2 minutes), and 100% alcohol (twice for 5 minutes each). The slides were cleared with xylene (three times, 5 minutes each), and neutral gum was applied to the center of the tissue on the slide. The cover glass was gently placed over the sample. The prepared slides were examined and photographed using the 3D Histech Digital Pathology System (Budapest, Hungary).

### Quantitative real-time polymerase chain reaction (qRT-PCR)

Total RNA was extracted from tissue samples using Trizol reagent (Ambion, USA), and mRNA was reverse transcribed to cDNA using the cDNA Synthesis SuperMix (TransGen Biotech, Beijing). Real-time PCR was performed using a Real-Time PCR system (ROCHE, Lightcycler 96, Switzerland) with the following thermal cycling conditions: initial denaturation at 95°C for 30 seconds, followed by 40 cycles of denaturation at 95°C for 5 seconds and annealing/extension at 60°C for 30 seconds. mRNA expression levels were normalized to the housekeeping gene GAPDH.

The fold-change in the expression of each target mRNA relative to GAPDH was calculated using the CT (2-ΔΔCT) method. Experiments were repeated at least three times, and the resulting data were statistically analyzed. The primer sequences are provided in Table 1.

### Immunohistochemistry

The expression of MMP2, MMP3, MMP7, MMP12, MMP13 and MMP19 in CC samples and BCL samples was analyzed by immunohistochemistry. After routine sectioning, the slides were dewaxed, dehydrated by gradient alcohol, blocked and inactivated by endogenous peroxidase, repaired by an antigen, and blocked by goat serum. Sections were incubated with the following primary antibodies at 4°C: MMP2 antibody (380817, Zenbio, 1:100 dilution), MMP3 antibody (340612, Zenbio, 1:100 dilution), MMP7 antibody (10374-2-AP, Proteintech, 1:200 dilution), MMP12 antibody (22989-1-AP, Proteintech, 1:200 dilution), MMP13 antibody (820098, Zenbio, 1:100 dilution), MMP19 antibody (860629, Zenbio, 1:100 dilution). Labeled secondary antibody was added and the mixture was incubated at 37°C. Horseradish peroxidase labeling solution was added, the mixture was stained with DAB, counterstained with hematoxylin, conventionally dehydrated, made transparent, and observed with a microscope after mounting. Sample visualization was performed with a Motic EasyScan (Motic, China) and representative fields were captured with Motic DSAassistantPlus software.

Staining intensity was quantified using the ImageJ IHC Profiler plugin. Staining was scored as 0 (no stain), +1 (weak stain), +2 (moderate stain), and +3 (strong stain) based on intensity. The H-score was calculated by multiplying the percentage of cells with each staining intensity value. The final IHC score (0~3) was calculated as: (percentage of high positive × 3) + (percentage of positive × 2) + (percentage of low positive × 1). To ensure accuracy, at least ten random visual fields on each slide were analyzed, and the final score for one slide was the average of all visual fields. This approach provided a comprehensive assessment of staining intensity and MMP expression levels. GraphPad Prism 9 software was used for statistical analysis, with p-values < 0.05 considered significant and indicated with asterisks (*, < 0.05; **, < 0.01; ***, < 0.001; ****, < 0.0001). Comparisons between the CC and BCL groups were performed using a two-tailed Student's t-test.

### Statistical analysis

The correlation between TME-infiltrated immune cells and *MMP* phenotypic expression was assessed using Spearman and distance correlation analysis. Differences among three or more groups were compared using one-way analysis of variance and the Kruskal-Wallis test^35^. The SurvMiner R package determined the cutoff point for dataset subgroups based on the correlation between the MMPscore and patient survival. The MMPscore was split using the "SURV-Cutpoint" function, dividing patients into high and low MMPscore groups based on the log-rank statistic. Prognostic analysis was conducted using Kaplan-Meier survival curves, and the significance of differences was determined using the log-rank test. The predictive value of the MMPscore for different tumor immunotherapies was evaluated using the Tumor Immune Dysfunction and Exclusion (TIDE) database.

The specificity and sensitivity of the MMPscore were assessed using the receiver operating characteristic (ROC) curve, and the area under the curve (AUC) was quantified using the PROC R software package (https://tide.dfci.harvard.edu/). Chromosomal copy number variation atlases for the three *MMP* phenotypes across 23 pairs of chromosomes were created using the RCIRCOS R package. p-values were analyzed from scRNA-Seq datasets of 20,938 cells, with a *p*-value < 0.05 considered statistically significant. All data processing was conducted using R3.6.1 software.

## Results

### Single-cell transcription mapping and cell typing of the CC matrix

**Figure 1:**
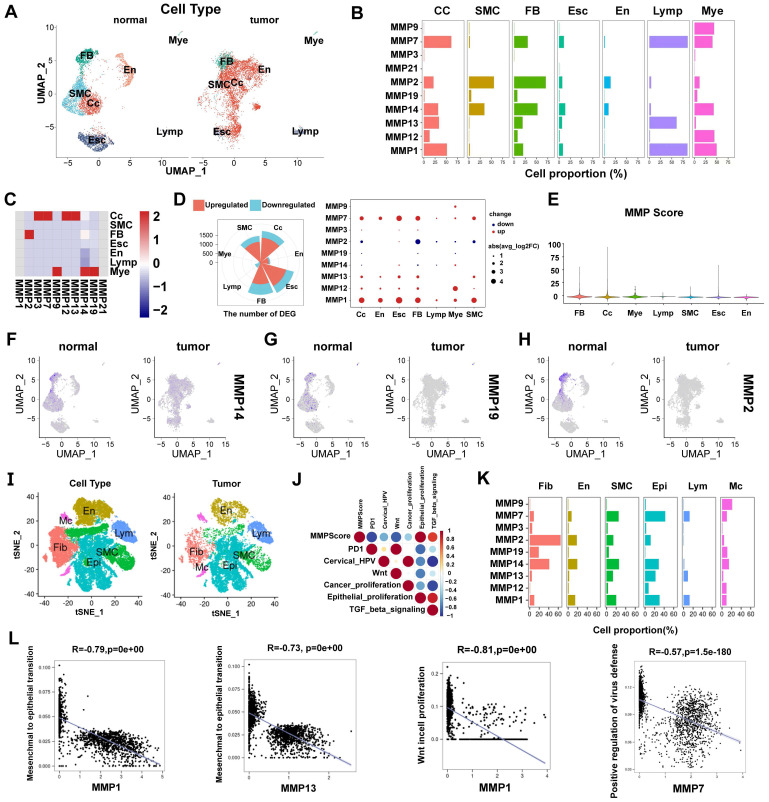
** (A)** Distribution and expression profiles of *MMPs* across individual cell types in the normal and CC. UMAP of seven cell types. **(B)** Percentage of *MMPs gene* in each cell type. **(C)** Heatmap of *MMPs* gene for seven cell types. **(D)** Differential expression of *MMPs* in different cell types of CC patients compared with control samples; the size of the dots indicated the average multiple of difference, and the color of the dots indicated up-regulated (red) or down-regulated (purple). **(E)**MMPscore in all cell clusters of individuals. Distribution and expression profiles of *MMP* across individual cell types in all the individuals. **(F-H)** The re-clustering showed distinct *MMPs* meant expression levels in all cell clusters in tumor tissue versus normal tissue. **(I)** tSNE of six cell types. **(J)** Correlation plot between *MMPs* score and pathway activities in CC tissue; the gradient of the dot represented the magnitude of the correlation. **(K)** Percentage of *MMPs gene* in each cell type. **(L)** Spearman correlation between *MMP-1* (*MMP-13*) and EMT. Spearman correlation between *MMP-7* and defense response to virus by host. Spearman correlation between *MMP-1* and *Wnt* pathway in regulation of cell proliferation.

To understand the cellular diversity and molecular features of cervical tissues in patients with CC, we have analyzed scRNA-seq datasets of 20,938 cells from patients with CC and BCL. Of these cells, 10,395 were obtained from the tumor samples that comprised seven cell types, including Myeloid cells, lymphocytes, endothelial cells, endometrial stromal cells, fibroblasts, smooth muscle cells, and CC cells, which were compared with those of the BCL group and annotated using classical marker genes (Figure 1A and S1A). The heatmap showed marker genes (Figure S1B and S1C). To understand the proportion of *MMP* molecules in each cell type, we used the bar graph to reveal the percentage of *MMPs gene* expression in each cell type. We found that *MMP-1*, *MMP-7*, *MMP-13* and *MMP-14* had a high proportion of diverse cell types (Figure 1B). We also concluded conclusion that *MMP-3*, *MMP-7*, *MMP-13* and *MMP-14* were up-regulated in CC cells in individuals and *MMP-9, MMP-14* and *MMP-19* were up-regulated in Myeloid cells by employing the heatmap (Figure 1C). To elucidate the mechanism of *MMP* regulation in diverse cell types in CC, we identified DEGs in individuals between MMPs high-expression and low-expression groups, finally it was found *MMP-1*, *MMP-12*, *MMP-13* and *MMP-7* were upregulated, but *MMP-14*, *MMP-19*, *MMP-2*, and *MMP-21* were downregulated in diverse cell types (Figure 1D), suggesting biochemically redundant members of the *MMP* family may have intricate interplay in tumor progression. To further explain the relationship between the expression of five *MMPs* in diverse cell types, we performed the re-clustering and Uniform Manifold Approximation and Projection (UMAP) dimensionality reduction profiles of *MMPs* in the CC and BCL groups. We found that the expressions of *MMP-14*, *MMP-19* and *MMP-2* were upregulated in BCL individuals compared to CC (Figure 1F-H), during which fibroblasts and myeloid cells displayed abundant *MMP* expression, indicating that the *MMPs gene* set could participate in inhibits tumor progression of CC. To identify whether *MMP* molecules can be linked to a favorable prognosis in CC, we then put *MMPs* as a gene module to calculate the score using AddModuleScore methods. The violin plot illustrated the fibroblasts, CC, and Mye cell had a higher score than the other cell types, consistently showing that the MMPscore was associated with immune responses in CC (Figure 1E).

To investigate the association between the classical CC pathways and MMPscore more accurately, we analyzed another scRNA-seq datasets from three CC and three BCL (Figure 1I), which encompassed 50,014 cells from patients with CC and BCL that consisted of seven cell types, including macrophage, lymphocytes, Neutrophils, endothelial cells, fibroblasts, smooth muscle cells, and epithelial cells (CC cells) (Figure 1J). The CC patient group had more abundant epithelial cells, macrophages and lymphocytes (Figure S1D). To understand the proportion of *MMP* molecules in each cell type, the bar graph showed the percentage of *MMP-1*, *MMP-2*, *MMP-7*, *MMP-13* and *MMP-14* had a high proportion of diverse cell types (Figure 1K). By identifying DEGs between *MMP* high-expression and low-expression groups, *MMP-1*, *MMP-3*, *MMP-12*, *MMP-13* and *MMP-7* were upregulated, but *MMP-2*, *MMP-14* and* MMP-19* were downregulated in diverse cell types (Figure S1E-G). We also put *MMPs* as a gene module to calculate the score using AddModuleScore methods and to define scores separately. Then we found that fibroblasts, CC, and Mye cell also had a higher score, suggesting that *MMP*-related genes played function more in these cell types (Figure S2A). A higher score for CC patients than for BCL may indicate a potential association of *MMP*-related genes and CC. To probe the association between the *MMP*-regulation and progression of CC, we used the functional enrichment analyses based on the GSEA database, and we performed the correlation between MMPscore by AUCell and classical pathways in CC to explore the influence of *MMP* regulators on CC pathways. Notably, we found MMPscore was upregulating *TGF-β* pathway (R > 0.6) and epithelial cell proliferation (R > 0.8), but downregulating PD-1 (R < -0.8), HPV copy number (R < -0.4), *Wnt* pathway (R < -0.8) and CC cell proliferation (R < -0.2) in CC patients (Figure 1J). To explore whether *MMPs* were associated with *Wnt* pathway in regulation of cell proliferation, with defense to virus by host and epithelial-mesenchymal transition (EMT). A correlation analysis combined with the MetaCell algorithm (K = 30) revealed that *MMP-1* (R = -0.79, *p* < 0.001), *MMP-7* (R = -0.69, *p* < 0.001) and *MMP-13* (R = -0.73, *p* < 0.001) were negatively associated with EMT in CC. *MMP-1* (R = -0.8, *p* < 0.001), and *MMP-7* (R = -0.81, *p* < 0.001) and *MMP-13* (R = -0.76, *p* < 0.001) were negatively associated with *Wnt* pathway in regulation of cell proliferation. Scatter plot showed that *MMP-7* (R= -0.57, *p* < 0.001) and *MMP-13* (R = -0.55, *p* < 0.001) were negatively associated with positive regulation of defense response to virus by host in CC (Figure 1L and Figure S1H-K). These results indicated that cell-type-specific up-regulation of *MMP* molecules was in normal cervical epithelial cell proliferation and inhibition to HPV infection, *Wnt* signaling, CC cell proliferation, EMT and PD1 in tumor proteins which controlled CC progression.

### *MMP* genetic variation and expression landscape in CC

**Figure 2:**
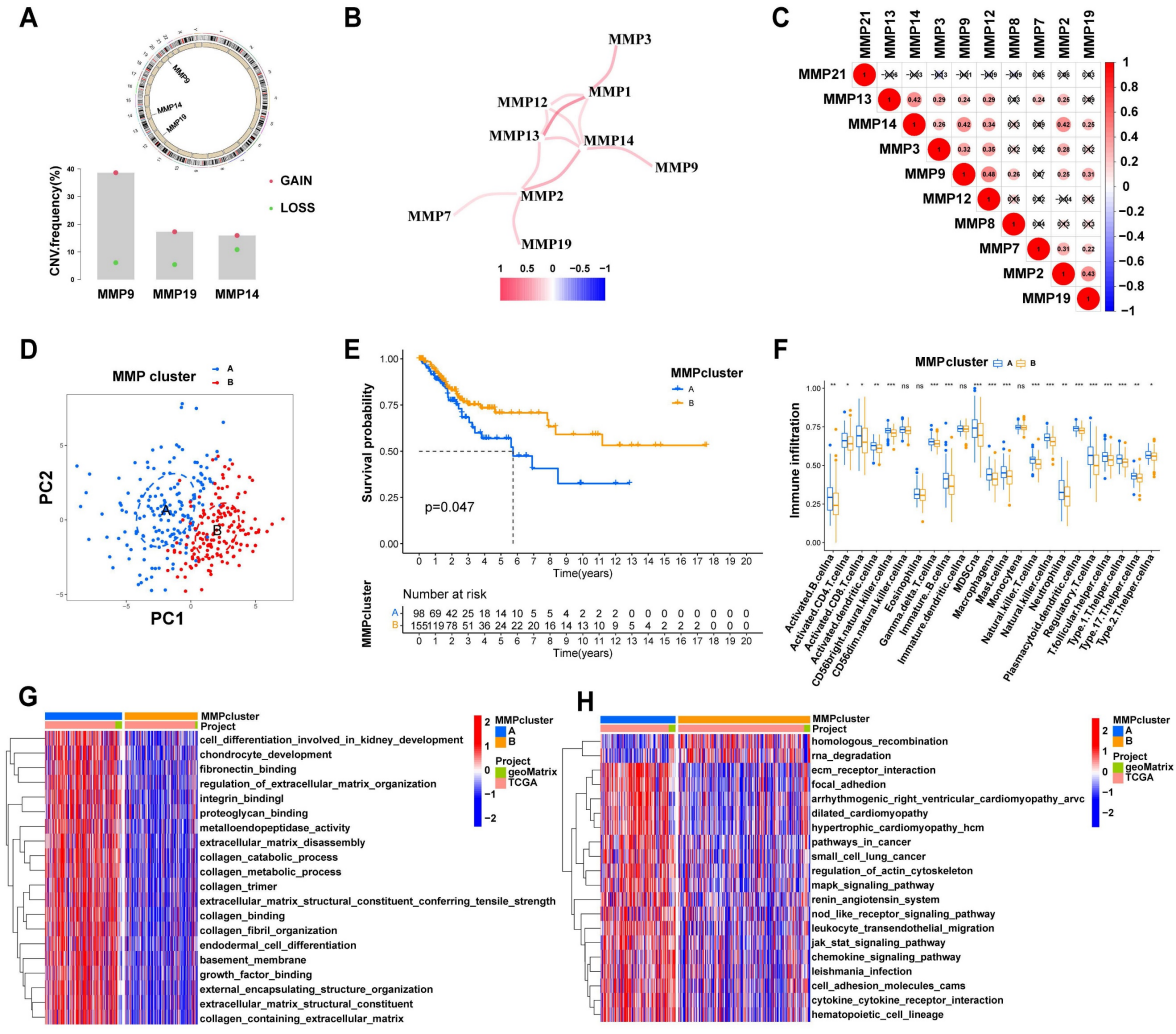
** (A)** 1. The location of CNV alteration of *MMPs* on 23 chromosomes. 2. The CNV variation frequency of *MMPs* in TCGA. The height of the column represented the alteration frequency. The deletion frequency, green dot; the amplification frequency, red dot. **(B)** The interaction of expression on 9 *MMPs* in CC. **(C)** Correlation plot of 10 *MMPs*. The positive correlation was marked with red, and negative correlation was marked with blue. The size of circle meant the absolute value of correlation coefficients. **(D)** Principal component analysis for the transcriptome profiles of *MMP* expression patterns, showing a remarkable difference on transcriptome between different modification patterns. **(E)** Survival analyses for the two *MMP* expression patterns based on 1649 patients with CC from TCGA cohorts including 98 cases in MMPcluster-A and 1551 cases in MMPcluster-B. Kaplan-Meier curves with Log-rank *p* value 0.047 showed a significant survival difference among two *MMP* expression patterns. The MMPcluster B displayed significantly better overall survival than MMPcluster-A. **(F)** The fraction of tumor-infiltrating lymphocyte cells in two *MMP* clusters by using the CIBERSORT algorithm. Within each group, the scattered dots indicated TME cell expression values. With the thick line of the median value, the bottom and top of the boxes were the 25th and 75th percentiles (interquartile range). * *p* < 0.05; ** *p* < 0.01; *** *p* < 0.001. **(G)** GSVA analyses showing the cellular component, molecular function and distinct biological processes in distinct MMPcluster expression patterns. The heatmap was used to visualize these biological processes with red activated pathways and blue inhibited pathways. **(H)** GSVA enrichment analysis showing the activation states of biological pathways in distinct *MMP* expression patterns. The heatmap was used to visualize these biological processes with red activated pathways and blue inhibited pathways.

In this study, we first summarize the prevalence of *MMP* CNV and somatic mutations in CC. Investigating CNV alteration frequency showed prevalent CNV alteration in 21 regulators of three *MMPs*, mostly focusing on copy number amplification (Figure 2A2). The chromosomal locations of *MMP* CNV alterations are shown in Figure 2A1. By investigating *MMP* mRNA expression levels in CC and BCL samples to determine whether the above genetic variations influenced *MMP* expression in patients with CC, we have found that CNV change may be the main factor leading to abnormal *MMP* expression. Compared with BCL tissues, *MMP-9* with amplified CNV demonstrated markedly higher expression in CC tissues (Figure S2B). There were significant differences in the expression of ten *MMPs* between patients with CC and BCL controls (Figure S2C). The correlation network diagram in Figure 2B depicts *MMP* interactions and further confirmed the ubiquitous correlation of the ten *MMPs*. We then examined the Pearson correlation between ten *MMPs* associated with CC by Spearman's correlation analyses, which observes a positive correlation among *MMPs* (Figure 2C). In TCGA cohort, we use the R package “ConsensusClusterPlus” to categorize a series of patients with different *MMP* expression patterns according to the expression of the ten *MMPs*. With final identification of two distinct modification patterns by unsupervised clustering (Figure S2D), we name these patterns MMPclusterA and -B that were supported by the principal component analysis results (Figure 2D, Figure S2E-I). Prognostic analysis of the two *MMP* expression subtypes reveals a prominent survival advantage within the MMPcluster-B expression pattern (*p* = 0.047) (Figure 2E). We further explored the different clinicopathological features and prognoses between the two groups (Figure S2K). The assessment of these data showed ten highly expressed *MMPs* in MMPcluster-A (Figure S2J). In addition, we also examined the different expression of four *MMP* types, and a significant correlation with overall survival prediction was seen in patients with CC (Figure S3A-D).

### Immune cell infiltration characteristics in the TME under different *MMP* expression patterns

The biological behaviors of various *MMP* expression patterns were analyzed by Kyoto Encyclopedia of Genes and Genomes (KEGG) enrichment. MMPcluster-A was significantly enriched in the matrix and carcinogenic activation pathways, like ECM receptor interactions, small cell lung cancer signaling pathways, cell adhesion, and MAPK signaling pathways, exhibiting significant aggregation during cell matrix catabolism. MMPcluster-B was mainly enriched in gene mutations, encompassing activation of homologous recombinant signaling pathways and RNA degradation signaling pathways (Figure 2G). Furthermore, *MMP* expression patterns greatly differed in terms of cell composition, molecular function, and biological processes. MMPcluster-A presented a generally enriched result, especially in collagen catabolism, metabolism, ECM catabolism, and constituent components, unlike MMPcluster-B (Figure 2H). Subsequent analyses indicated a higher degree of immune cell infiltration in the TME of MMPcluster-A, including activated T cells, CD56+ NK cells, MDSC, and plasmacytoid dendritic cells, while infiltration of CD56- NK cells, dendritic cells, and monocytes exhibited no clear difference between MMPcluster-A and -B (Figure 2F). Combination of KEGG, GO, and TME immune cell infiltration analysis showed that *MMP* expression patterns had obvious immune cell infiltration characteristics. Despite the significant immune cell infiltration, MMPcluster-A did not show a matching survival advantage (Figure 2E).

### Generation of *MMP* gene signatures and functional annotations

**Figure 3:**
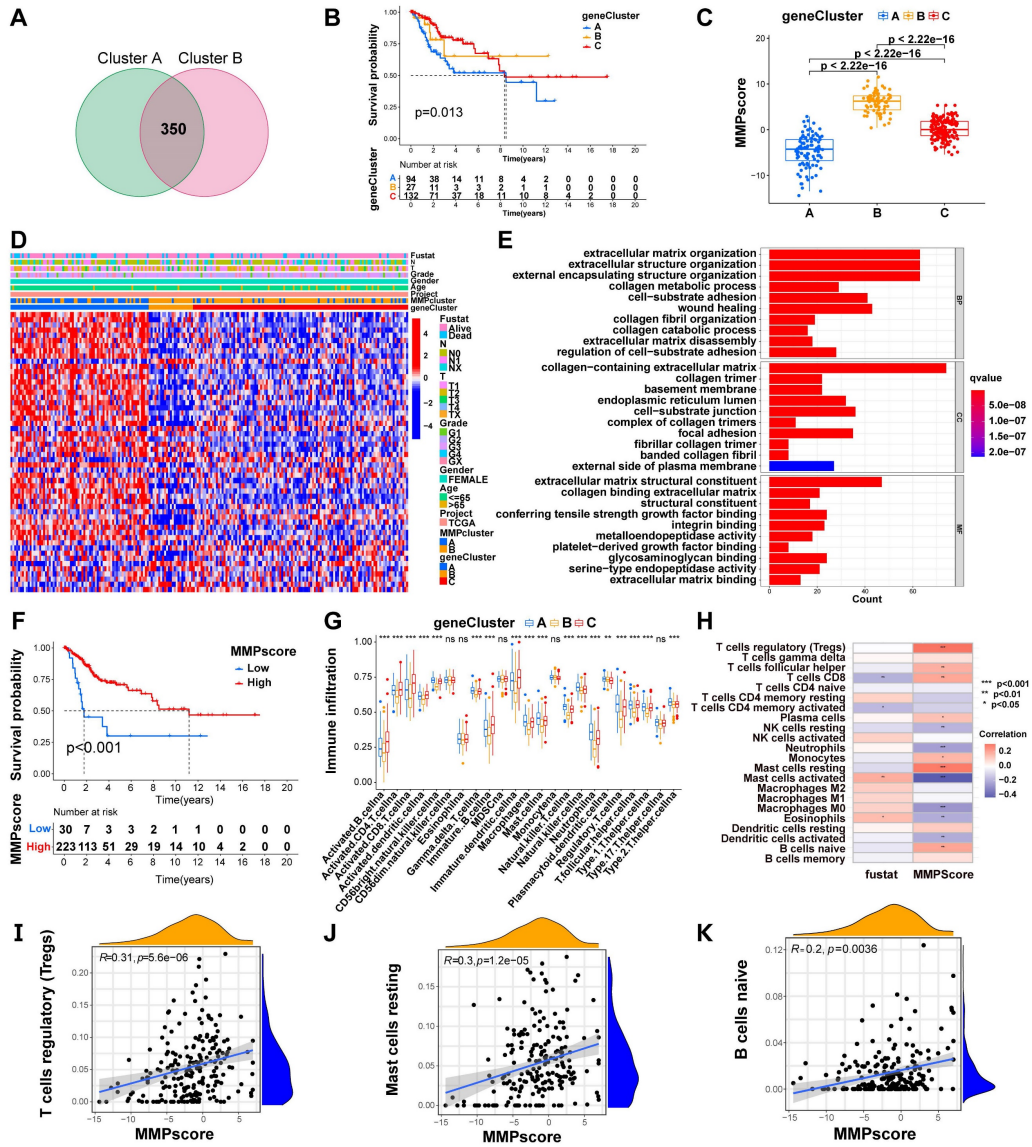
**(A)** 350 *MMP*-related DEGs between two MMPclusters were displayed in the Venn diagram. **(B)** Survival analyses for the three *MMP* expression patterns founded on 253 patients with CC from TCGA cohorts including 94 cases in MMPcluster-A, 27 cases in MMPcluster-B, and 132 cases in MMPcluster-C. Kaplan-Meier curves with Log-rank *p* value 0.013 showed an important survival difference among three *MMP* expression patterns. **(C)** Differences in MMPscore among three gene clusters. The Kruskal-Wallis test was utilized to compare the statistical differences between three gene clusters (*p* < 0.001). **(D)** Unsupervised clustering of overlapping *MMP* phenotype-related genes to classify patients into different genomic subtypes, termed as *MMP* gene cluster A-C, respectively. The gene clusters, MMPclusters, fustat, node, tumor, grade, gender and age were used as patient annotations. **(E)**The GO enrichment analysis was based on the overlapping *MMP* phenotype-related genes. The color bar represented the *p* values. BP, Biological Process; CC, Cellular Component; MF, Molecular Function. **(F)** Kaplan-Meier curves for high and low MMPscore patient groups in TCGA cohort. Log-rank test, *p* < 0.001. **(G)** The abundance of each TME infiltrating cell in three *MMP* gene clusters. The upper and lower ends of the boxes meant interquartile range of values. The lines in the boxes represented median value, and dots showed outliers. The asterisks indicted the statistical *p* value (* *p* < 0.05; ** *p* < 0.01; *** *p* < 0.001). **(H)** Correlation between different immune cells and fustat and MMPscore of CC patients. Red represented activated pathways and blue inhibited pathway. * *p* < 0.05; ** *p* < 0.01; *** *p* < 0.001. This was a Fig. Schemes following the same formatting. **(I)** Correlation between MMPscore and T cells regulatory (Tregs) in CC. R=0.31, *p* < 0.001. **(J)** Correlation between MMPscore and Mast cells resting in CC. R = 0.3, *p* < 0.001. **(K)** Correlation between MMPscore and B cells naive in CC. R = 0.2, *p* = 0.0036.

To further investigate the potential biological behavior of each* MMP* expression pattern, we identified two expression patterns (MMPcluster-A and MMPcluster-B) by unsupervised clustering. To prepare for the establishment of the MMPscore and visualize the heatmap of DEGs between MMPclusterA and -B, we obtained a total of 350 genes using DEG analyses (Figure 3A). Furthermore, through unsupervised clustering, DEGs were once again divided into three gene clusters: *MMP* gene cluster A-C (Figure S3E), representing the genetic differences in *MMPs*. To quantify the *MMP* landscape and facilitate the identification of key genes, PCA was used to compute the aggregate score of feature genes from three different *MMP* genome phenotypes, respectively. We obtained the sum of scores and defined them as the MMPscore. All TCGA patients were stratified into two groups with high or low MMPscore. As indicated from the prognostic analyses, the prognosis of the high score was better than that of the low score, 27 of 253 patients with CC clustered in gene cluster B, which was shown to be associated with a better prognosis. In contrast, 94 patients in gene cluster A exhibited poorer prognosis. An intermediate prognosis was observed in gene cluster C with 132 clustered patients. Prognostic analysis of the three *MMP* gene Cluster expression reveals a prominent survival advantage within the *MMP* gene cluster-B expression pattern (*p* = 0.013) (Figure 3B). In addition, we found that patients with advanced diseases were significantly associated with lower MMPscore, implying that these patients were characterized by a poorer clinical outcome in the MMPcluster-A modification pattern (*p* < 0.05) (Figure 3D), which suggested the two distinct *MMP* expression patterns in CC. We observed that *MMP* gene clusters A and B were consistent with the above expression patterns, whereas tumors in *MMP* gene cluster C exhibited poorer differentiation. Patients with clinically advanced diseases were characteristic of *MMP* gene cluster A, consistent with the above results (Figure 3D). By performing ontology enrichment analyses of different *MMP* gene clusters, we found that they were significantly enriched in ECM, collagen, and extracellular structural tissues.

Considering the individual heterogeneity of *MMP* expression (Figure 3E), we quantified *MMP* expression in individual tumor cells by calculating the MMPscore. Compared with the other clusters, MMPcluster-A showed a significantly lower MMPscore, while MMPcluster-B displayed a high median score (*p* < 0.05) (Figure S3F). The Kruskal-Wallis test implied crucial differences in MMPscore between *MMP* gene clusters (A vs B, *p* < 0.001; A vs C, *p* < 0.001; B vs C, *p* < 0.001). Gene cluster A had the lowest median score, while gene cluster B had the highest median score (Figure 3C). Consistent with the above analysis, this suggested that a high MMPscore may be strongly associated with immune activation, whereas a low MMPscore may be linked to stromal activation. Next, we sought to further determine the importance of the MMPscore in predicting patient prognosis. Patients were divided into low and high MMPscore groups. Patients with high MMPscore showed a significant survival benefit (*p* < 0.001), approximately twice that of patients with low MMPscore (Figure 3F). Besides, we found extremely different immune cell infiltration in the three gene clusters (Figure 3G). Further single-sample GSEA revealed that different MMPscore were significantly associated with high and low levels of immune infiltration in tumor tissues (Figure 3H and Figure S3G). The high MMPscore group was dominated by immune cell activation that was closely correlated with prognosis (Figure 3H). The above results strongly displayed that a high MMPscore was associated with increased immune activation. The MMPscore allowed for a better assessment of the *MMP* expression patterns of individual tumors and further assessed the TME infiltration characteristics of tumors to distinguish between true and false TME immune infiltration.

### Role of *MMP* expression patterns in immunotherapy

**Figure 4:**
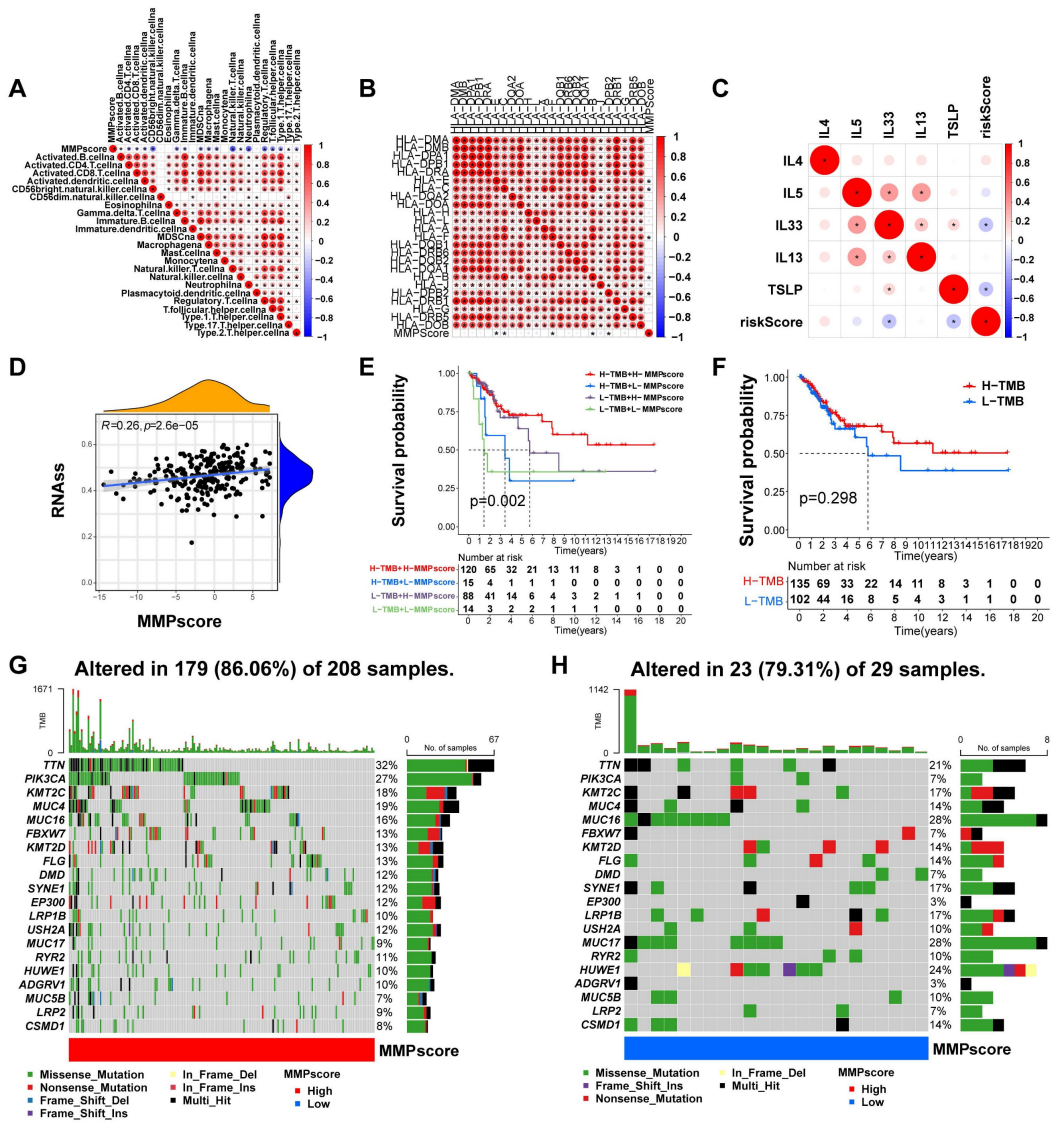
**(A)** Correlation between MMPscore and TME infiltrating cells. The color and the values indicate the Spearman correlation coefficient. (* *p* < 0.05; ** *p* < 0.01; *** *p* < 0.001). **(B)** Correlation between MMPscore and human leukocyte antigens in CC. The color and the values represented the Spearman correlation coefficient. The asterisks indicated a statistically vital p-value calculated using Mann-Whitney U test (* *p* < 0.05; ** *p* < 0.01; *** *p* < 0.001). **(C)** Correlation between MMPscore and Interleukins in CC. The color and the values represented the Spearman correlation coefficient. The asterisks indicated a statistically significant *p*-value calculated using Mann-Whitney U test (* *p* < 0.05; ** *p* < 0.01; *** *p* < 0.001). **(D)** Correlation between RNAss and MMPscore (R = 0.26, *p* < 0.05). **(E)** Survival analyses for patients with H-TMB+H-MMPscore, H-TMB+L-MMPscore, L-TMB+H-MMPscore and L-TMB+L-MMPscore using Kaplan-Meier curves. H, high; L, Low; TMB, Tumor Mutational Burden (*p* = 0.002, Log-rank test). The H-TMB+H-MMPscore showed much better overall survival than the other three *MMP* patterns. **(F)** Survival analyses for patients with high-Tumor Mutational Burden and low-Tumor Mutational Burden by Kaplan-Meier curves. H, high; L, Low; TMB, Tumor Mutational Burden. (*p* = 0.298, Log-rank test). **(G-H)** Mutational landscape of genes in TCGA stratified by high MMPscore **(G)** versus low MMPscore **(H)** subgroups. With each column representing individual patients, the upper bar plot showed TMB, and the right bar plot displayed the mutation frequency of each gene in separate MMPscore groups.

To better characterize the *MMP* immune profile and test the correlation between immune cells and MMPscore, we examined the specific correlation between each TME-infiltrating cell type and high and low *MMP* expression, which showed a tight correlation (Figure 4A). Our study implied that TME immune cell infiltration was significantly increased in tumors with high MMPscore, which meant a more significant positive correlation with T cell subsets, mast cells and B cells (T cells regulatatory, R = 0.31, *p* < 0.001; Mast cells resting, R = 0.3, *p* < 0.001; B cells naive, R = 0.3, *p* < 0.001; Mast cells activated, R = -0.43, *p* < 0.001; Dendritic cells activated, R = -0.21, *p* = 0.0022; Monocytes, R = 0.17, *p* = 0.016; Neutrophils, R = -0.24, *p* = 0.00057; NK cells resting, R = -0.19, *p* = 0.0059; Plasma cells, R = 0.14, *p* = 0.043; T cells CD8, R = 0.2, *p* = 0.004; Macrophages M0, R = -0.27, *p* < 0.001; T cells follicular helper, R = 0.18, *p* = 0.0098) (Figure 3I-K and Figure S3H-P). The investigation about the correlation between MMPscore and adhesion molecules, as well as between HLA molecules and interleukins, showed that MMPscore correlated significantly with immune checkpoints (Figure S3G and Figure 4B-C) with the most significant correlation of CD44 and TNFSF9 (CD44, R = 0.46, *p*<0.001; HLA-E, R = -0.18, *p* = 0.0044; TSLP, R = -0.27, *p* < 0.001; TNFSF9, R = -0.36, *p* < 0.001; IL33, R = -0.17, *p* = 0.0057; HLA-C, R = -0.15, *p* = 0.014) (Figure S4A-F). MMPscore can predict the strength and weakness of immune function with immunotherapy performed and evaluated for immune checkpoints. In addition, we found that different types of HLA also correlated with MMPscore (Figure 4B), with HLA-E and HLA-C the most prominent (Figure S4B and Figure S4D). For immune regulation, we found a correlation between MMPscore and interleukins (Figure 4C), with TLSP and IL-33 of more value (Figure S4C and Figure S4E). MMPscore also had predictive value in evaluating immune escape. The mRNA transcriptome showed differences with various MMPscore and correlated importantly with immune-related biological pathways (R = 0.26, *p*<0.001) (Figure 4D). The expression of *MMP* may be involved in immune pathways and activation or inhibition. CD47 increased immune escape, and a high MMPscore was associated with low immune escape in a study examining the effect of MMPscore on immune checkpoint blockade therapy (Figure S4G). These results showed that tumors with high *MMP* expression showed significantly correlated immune activation pathways.

### Characterization of *MMP* expression in TCGA molecular subtypes and tumor somatic cell mutations

TCGA constructed a comprehensive molecular landscape for CC and investigated the predictive ability of MMPscore in CC prognosis in patients with different tumor mutational burden, found that survival prognosis was not significantly related to high or low tumor loads (*p* = 0.298) (Figure 4F), however, high or low tumor mutational burden combined with the high MMPscore group had a better survival prognosis than those combined with the low MMPscore group (*p* = 0.002) (Figure 4E). We counted 179 *MMP* mutations in 208 CC samples with a mutation frequency of 86.06%, of which *TTN* showed the highest mutation frequency followed by *PIK3CA*, and other genes also displayed varying degrees of mutation (Figure 4G). The somatic mutation distribution differed between high and low MMPscore in the cohort (Figure 4G and 4H): the low MMPscore group exhibited a wider range of tumor mutations, compared with the high MMPscore group. The rates of the 2nd and 14th most significant mutated genes were 27% and 7%, and 9% and 28%, respectively. The above analyses indicated that a high MMPscore was associated with long-term survival and durable clinical benefit. The combination of MMPscore and tumor mutational burden could more accurately determine prognosis in different patient cohorts, and the predictive advantage of ROC curve assessment was reflected in the survival prognosis of patients with CC (Figure S4H).

### Clinical features of *MMP* expression patterns

**Figure 5:**
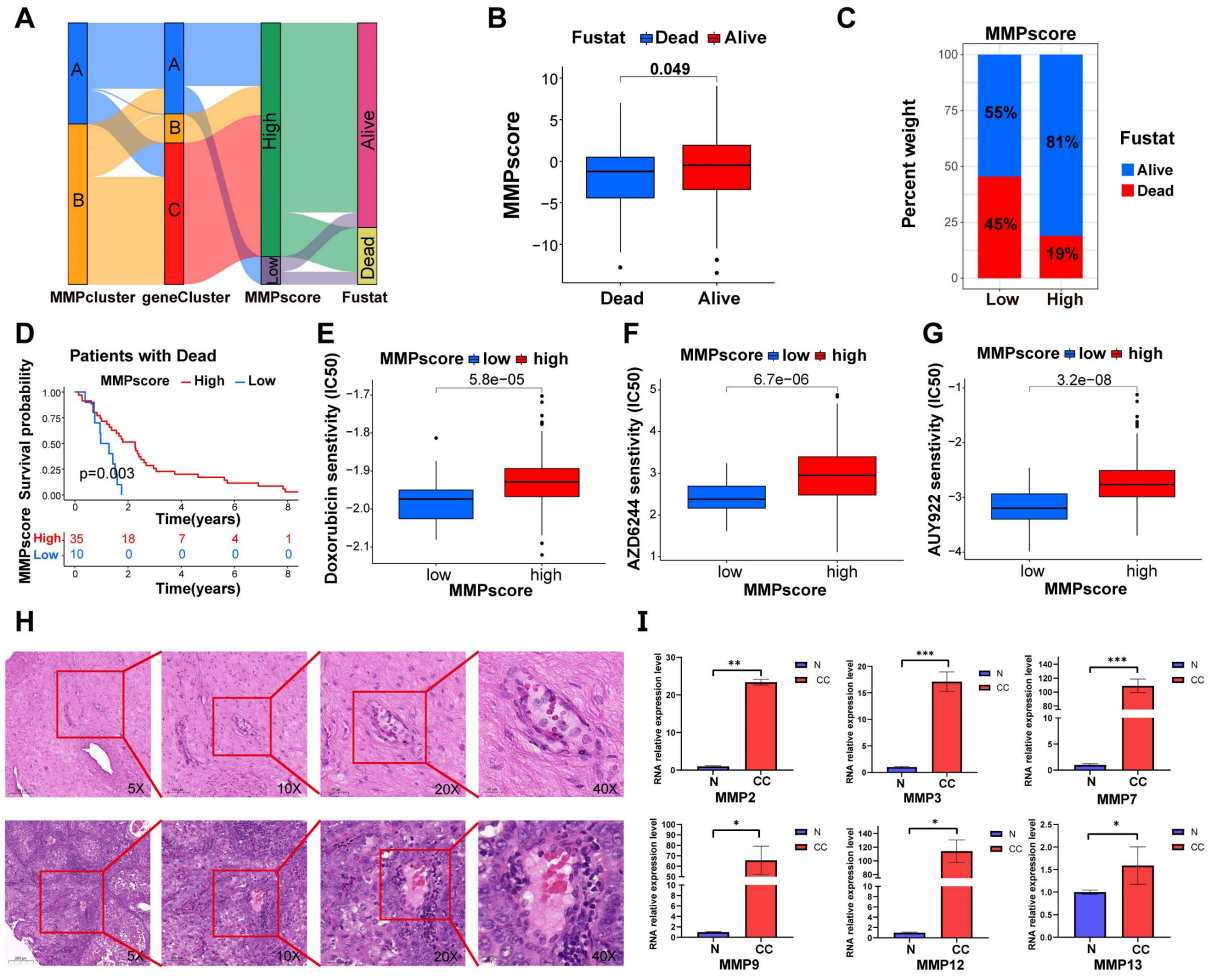
** (A)** Sankey diagram demonstrating the relationship between MMPclusters, geneclusters, MMPscore and fustat.** (B)** Differences in MMPscore among distinct fustat clinical response groups. **(C)** The proportion of patients with fustat in the low or high MMPscore group. **(D)** Kaplan-Meier curves for high and low MMPscore patient groups in the dead patients. Log-rank test, *p* = 0.003. **(E-G)** The correlation between the high and low expressions of* MMP* and the semi-inhibited concentration sensitivity of various drugs. Drugs: Doxorubicin, AZD6244, and AUY922. **(H)** HE staining was performed to observe pathological changes of cervix tissue in normal and CC group. (A, normal, B, CC; magnification: 5x, scale bar = 200µm; 10x, scale bar = 100µm; 20x, scale bar = 50µm; 40x, scale bar = 20µm). **(I)** Expression levels of mRNA of *MMPs* in CC and control. The mRNA expression levels of *MMP-2*, *MMP-3*, *MMP-7*, *MMP-9*, *MMP-12* and *MMP-13* in patients with CC or controls were measured by RT-qPCR. *GAPDH* were used as a loading control. Data were founded on the mean ± SD of triplicate independent experiments. *p* values were obtained by Student's t test. (CC, n = 3; Controls, n=3; *, *p* < 0.05; **, *p* < 0.01; ***, *p* < 0.001; ns, nonsignificant.).

Consistent with the above findings (Figure 5A), gene clusters B and C both showed high* MMP* expression and better survival outcomes, while gene cluster A, containing both high and low *MMP* expression cases, exhibited poorer survival outcomes, approximately 50% of patients with a prognosis of death. The above results again suggested that *MMP* expression profiles played a non-negligible role in shaping different TMEs. Next, we used MMPscore to systematically evaluate CC in terms of its clinical characteristics, including age, weight, smoking, clinical stage, and prognosis (Figure 5B-D, S4I-Q and Figure S5A-I). Once again, the high MMPscore (*p* = 0.003) was dominated by survival outcomes in the Fustat survival prognosis (Figure 5B-D). In addition, high MMPscore also correlated significantly with TNM stage, especially in patients with Nx (*p* = 0.006), T2 (*p*<0.001), and G3 (*p* = 0.006) stages of better survival prognosis (Figure S4J-L and Figure S5B, 5D, 5F), which showed better assessment in tumor infiltration and metastasis. We also found that the MMPscore could indicate sensitivity to more than ten drugs, including nilotinib and Adriamycin. It can be inferred that high MMPscore can make drug response more sensitive (Doxorubicin, *p* < 0.001; AZD6244, *p* < 0.001; AUY922, *p* < 0.001; AS601245, *p* < 0.001; ATRA, *p* < 0.001; AMG.706, *p* < 0.001; Nilotinib, *p* < 0.001; WO2009093972, *p* < 0.001; AZD.0530, *p* < 0.001; A.770041, *p* < 0.001) (Figure 5E-G and Figure S5J-P).

### MMP expression is generally increased in CC tissue

**Figure 6:**
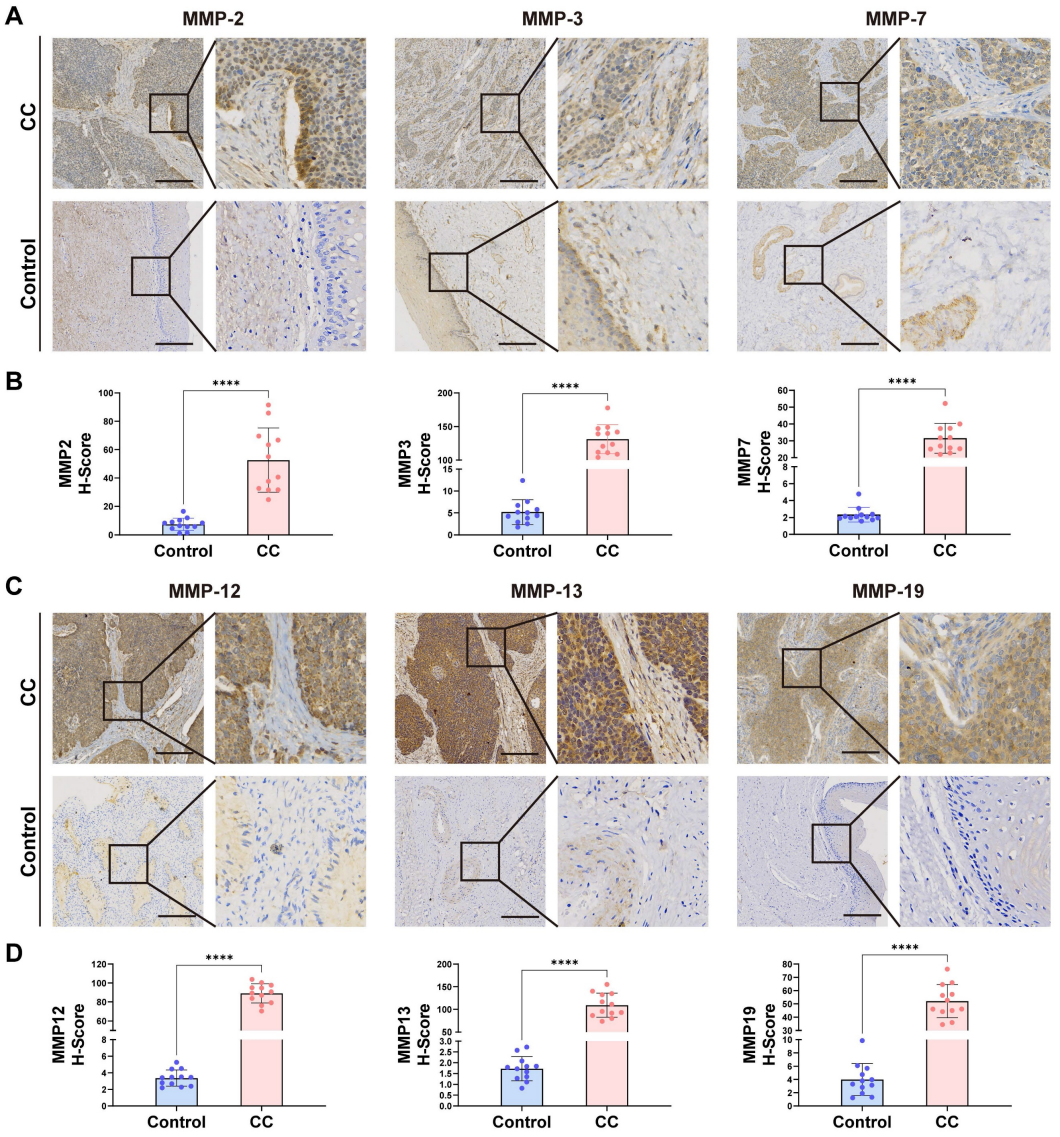
** (A-D)** Immunohistochemical analysis was performed on cervical tissues from control patients (n = 12) and those with CC (n =12). Representative images and data are shown. Scale bars: 200.0 µm(10X). *, *p* < 0.05; **, *p* < 0.01; ***, *p* < 0.001; ****, *p* < 0.0001.

To confirm whether the expression of the *MMP* gene set was generalized at the molecular level in tissues of patients with CC, we conducted HE and qRT-PCR experiments on cervical tissues from three cases of CC and three healthy individuals. As listed in Table S1, the six subjects enrolled in this study were divided into the CC group (three patients with CC receiving surgery or other treatments) and the non-CC group (three subjects receiving abdominal hysterectomy for uterine leiomyoma or prolapse of uterus). The age distribution, the parity, and times of pregnancy showed no significant group differences. HE staining revealed cell polarity disorder, increased mitotic Figs, and abnormal cells breaking through the basal layer in CC tissues, which was consistent with the diagnosis of CC. HE staining of cervical tissue in the BCL group conformed to normal cervical pathological characteristics (Figure 5H). We observed that the expression of *MMP-2*, *MMP-3*, *MMP-7*, *MMP-9*, *MMP-12*, and *MMP-13* genes in CC tissue was generally higher than that in BCL tissue (Figure 5I). qRT-PCR analysis displayed that the mRNA expression of *MMP-2*, *MMP-3*, *MMP-7*, and *MMP-9* in the cervical tissue of patients with cancer was significantly greater than that in the control (*p* = 0.005, *p* < 0.001, *p* < 0.001, and *p* = 0.038, respectively). mRNA expression levels of *MMP-12*, *MMP-13*, *MMP-14*, and *MMP-19* in the cervical tissue of patients with cancer were greater than that observed in the control (*p* = 0.045, *p* = 0.025, *p* = 0.003, and *p* = 0.025, respectively) (Figure 5I). We screened patients for immunohistochemical experiments using the same criteria as HE, and the patient information is shown in Table S2, Similarly, our immunohistochemical results also indicated that the tissue expression levels of MMP-2, MMP-3, MMP-7, MMP-12, MMP-13, and MMP-19 were higher than those of the control (*p* < 0.0001, respectively) (Figure 6A-D).

## Discussion

*MMPs* regulate cancer progression by regulating angiogenesis, invasion, and immune escape, but their potential for CC diagnosis and prognosis has not been studied considering the overall *MMPs* expression pattern 36.* MMPs* can bind to the cancer cell surface, act on all stages of cancer, and are expressed in early tumor cells to facilitate ECM remodeling and release membrane-bound growth factors that prepare the microenvironment for tumorigenesis 37, 38. MMP-9 plays a role in a wide variety of cancers due to its effects on immune cell infiltration, and it has shown potential to determine prognosis. In addition, *MMP-1* and *MMP-2* have also been associated with CC prognosis 39-42.* MMP-7* can predict a more aggressive colon cancer phenotype and is inversely correlated with patient survival 43. *MMP-11* can be considered a potential tumor marker and therapeutic target for advanced prostate cancer 44. The mechanism underlying *MMP* effects in CC still requires further study. Recently, *MMP* has shown a strong evaluation ability in terms of clinical pathological characteristics, prognosis, and immune phenotype, which revealed *MMP* expression characteristics in the CC TME in this study.

Accumulating evidence indicates that the *MMP* expression profile plays an indispensable role in inflammation 45, immunity, and inhibiting tumor progression, especially in the development and progression of digestive tract tumors, but there is no related study concerning CC. Furthermore, most current studies focus on a single TME cell type or a single protease, and the overall TME infiltration characteristics mediated by the combined effects of multiple *MMPs* have not been comprehensively recognized. We used the scRNA-seq dataset composed of CC patients and healthy individuals to find that *MMP* exhibited a cell type specific expression pattern in cervical tissue of cancer at the single cell level. This study revealed two *MMP* expression patterns based on 10 *MMPs*, which have shown distinctly different TME cell infiltration characteristics. MMPcluster-A is a type of immune rejection characterized by innate immune cell infiltration and stromal activation, as well as an immunoinflammatory phenotype characterized by adaptive immune cell infiltration and immune activation. In contrast, MMPcluster-B was activated by mutations. The immune rejection phenotype comprises non-inflamed tumors, while immune inflammatory phenotypes, referred to as hot tumors, manifest as massive immune cell infiltration in the TME 46-48. Although the immune rejection phenotype included a large number of immune cells, they remained in the stroma surrounding the tumor cell nests, rather than penetrating their parenchyma. The stroma can be confined to the tumor envelope or penetrate the tumor itself, making immune cells appear truly inside the tumor 49-51. More importantly, we found that MMPcluster-A exhibited a distinct stromal activation status, combined with TME cell infiltration features in each cluster. Patient prognosis opposed expectations; therefore, we speculated that stromal activation in MMPcluster-A inhibited the antitumor effect of immune cells. Significant prognostic differences existed between the two clusters, confirming the reliability of our immunophenotype classification for different *MMP* expression patterns. Therefore, fully exploring the TME cellular infiltration characterization induced by different *MMP* expression patterns demonstrated that MMPcluster-A could further lead to poor prognosis through the function of suppressed immune cells. In addition, comparing *MMP* genetic and expression alterations between CC tissues and normal tissues showed a certain heterogeneity, indicating that *MMP* expression imbalance may play an important role in CC occurrence and progression. Our seminal exploration of the overall *MMP* expression pattern role in TME infiltration in CC will contribute to a deeper understanding of the mechanism of TME antitumor immune response and a more effective strategy for guiding immunotherapy.

In this study, *MMP* gene analysis identified three genomic subtypes that were also significantly associated with matrix activation and immune response, similar to the clustering results for *MMP* expression. This demonstrates that *MMP* expression has important implications for shaping different TMEs. Therefore, a comprehensive evaluation of *MMP* expression patterns will enhance our understanding of the characterization of TME cellular infiltration. However, previous analysis was mainly based on patient population and could not accurately predict the expression pattern in individual patients. Considering the individual heterogeneity of *MMP* expression, its pattern must be urgently quantified in single tumors. In this study, this deficiency was well compensated by constructing an *MMP* scoring system, evaluating the *MMP* expression pattern in CC patients, and visualizing property changes in individual patients. The expression pattern dominated by the MMPcluster-B expression signature exhibited a high MMPscore, suggesting that the MMPscore is a reliable and powerful tool to comprehensively assess *MMP* expression patterns in individual tumors that can be used to further determine the TME infiltration pattern, also known as the tumor immunophenotype. More importantly, the MMPscore showed good assessment ability in terms of patient clinical characteristics, including tumor differentiation level, mutation burden, pathological stage, body weight, age, and clinical prognosis, and it could guide clinical treatment. Comprehensive analysis showed that MMPscore was an effective indicator of biological prognosis in CC. Our MMPscore has shown excellent predictive power in CC precision immunotherapy utilizing the immune escape feature.

The heterogeneity of solid tumors and the TME has been well mapped. In this study, we elucidated the heterogeneity and tumor infiltration pattern in CC through different *MMP* expression patterns. Some studies have shown that the CC TME is heterogeneous 52, and we further analyzed its biological and transcriptomic heterogeneity at the single-cell level. Different CC cell markers identified tumor cell heterogeneity, as well as apparent heterogeneity in *MMP* expression, compared with tumor stromal cells, which suggested that different transcriptomes of individual cells might reflect their tumor biological characteristics, further demonstrating the possibility that *MMPs* are involved in and influence tumor progression. By using scRNA-seq, we identified all cell types in CC, including those of neutrophils, T cells, smooth muscle cells, trophoblast progenitors, trophoblast stem cells, megakaryocytic progenitors, endothelial cells, myoepithelial cells, Purkinje cells, myeloid cells, and fibroblasts, as well as 12 cell types of unknown significance. Further dimension reduction analysis showed that the proportion of each cell subtype differed significantly between cancer tissues and normal tissue stroma. GSEA of the marker genes of each cancer cell cluster revealed some association with the *MMP* family, further validating the cell identity and biological pathways described above and elucidating specific gene expression signatures in CC cell types while comparing the different cell infiltration in tumor tissues and showing their *MMP* enrichment. Single-cell transcriptomics explain the potential link between tumor cells and *MMPs* by identifying rare cell subpopulations. In addition, the effectiveness of MMPscore was also well validated in CC tissue. Therefore, we anticipate that these findings will provide important clues for the developing applications of the *MMP* family in CC.

Unfortunately, our data did not show a significant correlation between MMPscore and tumor mutational burden. However, it is worth noting that patients in high MMPscore group showed better prognosis regardless of high or low tumor burden, and then it was speculated that survival analysis by MMPscore combined with tumor mutational burden could yield more nuanced prognostic results. Our results showed that *MMP* expression plays a non-negligible role in shaping different stromal and immune TMEs, implying that it may influence the therapeutic efficacy of immune checkpoint blockade. In addition, MMPscore integration with various biomarkers, including PD-1 expression and stromal and immune TME, may lead to a more effective CC immunotherapy strategy, which should be verified by further experimental results. Moreover, the correlation between MMPscore and tumor stage, degree of invasion, and prognosis analysis did not show significant effect in all grades. The correlation between MMPscore and patient weight and age factors and its survival predictive effect were also not obvious. With the power of single-cell transcriptomics to identify cellular subsets and interpret links between tumor cells and endothelial cells, scRNA-seq is inherently limited to transcript-level measurements, so the functional implications of each population require further investigation.

Scholars performing pan-cancer analysis showed that *MMPs* had prognostic value only in clear cell renal cancer 36. This study elucidates the role of *MMPs* in cancer by developing an MMPscore scoring system, which may serve as an independent marker for predicting patient survival prognosis and provide new insights into CC immunotherapy. These new ideas may target MMP-related genes, reverse unfavorable TME cell infiltration characterization, and help develop novel drug combination strategies or novel immunotherapeutic agents in the future. We provide new ideas for improving patient clinical responses to immunotherapy, identifying distinct tumor immune phenotypes, and promoting personalized CC immunotherapy in the future. In conclusion, the MMPscore can be used in clinical practice to comprehensively evaluate the *MMP* expression pattern of individual patients and their corresponding TME cell infiltration characteristics, further determine the tumor immune phenotype, and guide more effective clinical practice.

## Conclusions

In summary, this work demonstrates a broad regulatory mechanism of the CC TME by the *MMP* expression landscape. Differences in *MMP* expression patterns are a non-negligible factor contributing to the heterogeneity and complexity of individual TMEs. The MMPscore exhibited a strong predictive function in CC patient survival analysis that could provide guidance for clinical work-up. Single cell transcriptomics used to investigate the intratumoral heterogeneity of CC at the cellular level validated the strong correlation between *MMPs* and tumorigenesis and revealed the biological nature of tumorigenesis. Comprehensive assessment of *MMP* expression patterns in individual tumors will enhance our understanding of the characteristics of cellular infiltration in the TME. The correlation between MMPscore and immune checkpoints and immune cells may provide strategies and directions for subsequent immunotherapy research.

## Supplementary Material

Supplementary figures and tables.

## Figures and Tables

**Table 1:** Primer sequences used in the study.

Genes	Primer nucleotide sequence (5′ to 3′)	
MMP-2	F: CCTACACCAAGAACTTCCGTCTG	R: GTGCCAAGGTCAATGTCAGGAG
MMP-3	F: CCTTTCCTGGCATCCCGAAGTG	R: GCCTGGAGAATGTGAGTGGAGTC
MMP-7	F: GAGGATGAACGCTGGACGGATG	R: AGGATCAGAGGAATGTCCCATACCC
MMP-8	F: GGAACGCACTAACTTGACCTACAGG	R: AACACTCCAGAGTTCAAAGGCATCC
MMP-9	F: CTGGTCCTGGTGCTCCTGGTG	R: CTGCCTGTCGGTGAGATTGGTTC
MMP-12	F: TGGACCTGGATCTGGCATTGGAG	R: TCGTGAACAGCAGTGAGGAACAAG
MMP-13	F: GGAGATGAAGACCCCAACCCTAAAC	R: CGGAGACTGGTAATGGCATCAAGG
MMP-19	F: CGGAGACTGGTAATGGCATCAAGG	R: CAAAGGGCAGACACTCGGAACAAG
GAPDH	F: GCTCTCTGCTCCTCCTGTTC	R: ACGACCAAATCCGTTGACTC

## Abbreviations

MMP: matrix metalloproteinase
TME: tumor microenvironment
CC: cervical cancer
scRNA-seq: single-cell RNA sequencing
qRT-PCR: quantitative Real-Time Polymerase Chain Reaction
HPV: human papillomavirus
TAM: tumor-associated macrophages
ECM: extracellular matrix
GEO: Gene Expression Omnibus
DEGs: differentially expressed genes
GO: Gene Ontology
GSEA: gene set enrichment analysis
TCGA: The Cancer Genome Atlas
CNV: copy number variation
ssGSEA: single-sample gene-set enrichment analysis
PCA: principal component analysis
HE: hematoxylin-eosin staining
TIDE: Tumor Immune Dysfunction and Exclusion
ROC: receiver operating characteristic
AUC: area under the curve
UMAP: Uniform Manifold Approximation and Projection
EMT: epithelial-mesenchymal transition
KEGG: Kyoto Encyclopedia of Genes and Genomes
IHC: immunohistochemical

## References

[1] Wang Z, Wang W, Yang A, Zhao W, Yang J, Wang Z (2021). Lower dietary mineral intake is significantly associated with cervical cancer risk in a population-based cross-sectional study. J Cancer.

[2] Bosch FX, Lorincz A, Muñoz N, Meijer CJ, Shah KV (2002). The causal relation between human papillomavirus and cervical cancer. J Clin Pathol.

[3] Shields TS, Brinton LA, Burk RD, Wang SS, Weinstein SJ, Ziegler RG (2004). A case-control study of risk factors for invasive cervical cancer among U.S. women exposed to oncogenic types of human papillomavirus. Cancer Epidemiol Biomarkers Prev.

[4] Kjellberg L, Hallmans G, Ahren AM, Johansson R, Bergman F, Wadell G (2000). Smoking, diet, pregnancy and oral contraceptive use as risk factors for cervical intra-epithelial neoplasia in relation to human papillomavirus infection. Br J Cancer.

[5] Siegel RL, Miller KD, Fuchs HE, Jemal A (2022). Cancer statistics, 2022. CA Cancer J Clin.

[6] Dyba T, Randi G, Bray F, Martos C, Giusti F, Nicholson N (2021). The European cancer burden in 2020: Incidence and mortality estimates for 40 countries and 25 major cancers. Eur J Cancer.

[7] Li K, Xu H, Wang S, Qin P, Liang B (2022). Disparities in the increases of cervical cancer incidence rates: observations from a city-wide population-based study. BMC Cancer.

[8] Noguchi T, Zaitsu M, Oki I, Haruyama Y, Nishida K, Uchiyama K (2020). Recent Increasing Incidence of Early-Stage Cervical Cancers of the Squamous Cell Carcinoma Subtype among Young Women. Int J Environ Res Public Health.

[9] Chen D, Zhang X, Li Z, Zhu B (2021). Metabolic regulatory crosstalk between tumor microenvironment and tumor-associated macrophages. Theranostics.

[10] Mao X, Xu J, Wang W, Liang C, Hua J, Liu J (2021). Crosstalk between cancer-associated fibroblasts and immune cells in the tumor microenvironment: new findings and future perspectives. Mol Cancer.

[11] Zhou J, Zhang S, Guo C (2021). Crosstalk between macrophages and natural killer cells in the tumor microenvironment. Int Immunopharmacol.

[12] Li C, Guo L, Li S, Hua K (2021). Single-cell transcriptomics reveals the landscape of intra-tumoral heterogeneity and transcriptional activities of ECs in CC. Mol Ther Nucleic Acids.

[13] Bu L, Baba H, Yoshida N, Miyake K, Yasuda T, Uchihara T (2019). Biological heterogeneity and versatility of cancer-associated fibroblasts in the tumor microenvironment. Oncogene.

[14] Patel AK, Singh S (2020). Cancer associated fibroblasts: phenotypic and functional heterogeneity. Front Biosci (Landmark Ed).

[15] Wu F, Fan J, He Y, Xiong A, Yu J, Li Y (2021). Single-cell profiling of tumor heterogeneity and the microenvironment in advanced non-small cell lung cancer. Nat Commun.

[16] Ahirwar DK, Charan M, Mishra S, Verma AK, Shilo K, Ramaswamy B (2021). Slit2 Inhibits Breast Cancer Metastasis by Activating M1-Like Phagocytic and Antifibrotic Macrophages. Cancer Res.

[17] Goswami KK, Bose A, Baral R (2021). Macrophages in tumor: An inflammatory perspective. Clin Immunol.

[18] O'Grady A, Dunne C, O'Kelly P, Murphy GM, Leader M, Kay E (2007). Differential expression of matrix metalloproteinase (MMP)-2, MMP-9 and tissue inhibitor of metalloproteinase (TIMP)-1 and TIMP-2 in non-melanoma skin cancer: implications for tumour progression. Histopathology.

[19] Kuivanen T, Jeskanen L, Kyllönen L, Isaka K, Saarialho-Kere U (2009). Matrix metalloproteinase-26 is present more frequently in squamous cell carcinomas of immunosuppressed compared with immunocompetent patients. J Cutan Pathol.

[20] Hernández-Pérez M, El-hajahmad M, Massaro J, Mahalingam M (2012). Expression of gelatinases (MMP-2, MMP-9) and gelatinase activator (MMP-14) in actinic keratosis and in in situ and invasive squamous cell carcinoma. Am J Dermatopathol.

[21] Lederle W, Hartenstein B, Meides A, Kunzelmann H, Werb Z, Angel P (2010). MMP13 as a stromal mediator in controlling persistent angiogenesis in skin carcinoma. Carcinogenesis.

[22] Kivisaari AK, Kallajoki M, Ala-aho R, McGrath JA, Bauer JW, Königová R (2010). Matrix metalloproteinase-7 activates heparin-binding epidermal growth factor-like growth factor in cutaneous squamous cell carcinoma. Br J Dermatol.

[23] Chan LP, Tseng YP, Wang HC, Chien CY, Wu CW, Wang LF (2022). Growth regulated oncogene-α contribute to EMT/MMPs pathway by binding its receptors in head and neck squamous cell carcinoma. Life Sci.

[24] Chuang HC, Su CY, Huang HY, Huang CC, Chien CY, Du YY (2008). Active matrix metalloproteinase-7 is associated with invasion in buccal squamous cell carcinoma. Mod Pathol.

[25] Roh MR, Zheng Z, Kim HS, Kwon JE, Jeung HC, Rha SY (2012). Differential expression patterns of MMPs and their role in the invasion of epithelial premalignant tumors and invasive cutaneous squamous cell carcinoma. Exp Mol Pathol.

[26] Pittayapruek P, Meephansan J, Prapapan O, Komine M, Ohtsuki M (2016). Role of Matrix Metalloproteinases in Photoaging and Photocarcinogenesis. Int J Mol Sci.

[27] Vosseler S, Lederle W, Airola K, Obermueller E, Fusenig NE, Mueller MM (2009). Distinct progression-associated expression of tumor and stromal MMPs in HaCaT skin SCCs correlates with onset of invasion. Int J Cancer.

[28] Sbardella D, Fasciglione GF, Gioia M, Ciaccio C, Tundo GR, Marini S (2012). Human matrix metalloproteinases: an ubiquitarian class of enzymes involved in several pathological processes. Mol Aspects Med.

[29] Wilkerson MD, Hayes DN (2010). ConsensusClusterPlus: a class discovery tool with confidence assessments and item tracking. Bioinformatics.

[30] Hänzelmann S, Castelo R, Guinney J (2013). GSVA: gene set variation analysis for microarray and RNA-seq data. BMC Bioinformatics.

[31] Charoentong P, Finotello F, Angelova M, Mayer C, Efremova M, Rieder D (2017). Pan-cancer Immunogenomic Analyses Reveal Genotype-Immunophenotype Relationships and Predictors of Response to Checkpoint Blockade. Cell Rep.

[32] Barbie DA, Tamayo P, Boehm JS, Kim SY, Moody SE, Dunn IF (2009). Systematic RNA interference reveals that oncogenic KRAS-driven cancers require TBK1. Nature.

[33] Sotiriou C, Wirapati P, Loi S, Harris A, Fox S, Smeds J (2006). Gene expression profiling in breast cancer: understanding the molecular basis of histologic grade to improve prognosis. J Natl Cancer Inst.

[34] Zeng D, Li M, Zhou R, Zhang J, Sun H, Shi M (2019). Tumor Microenvironment Characterization in Gastric Cancer Identifies Prognostic and Immunotherapeutically Relevant Gene Signatures. Cancer Immunol Res.

[35] Hazra A, Gogtay N (2016). Biostatistics Series Module 3: Comparing Groups: Numerical Variables. Indian J Dermatol.

[36] Gobin E, Bagwell K, Wagner J, Mysona D, Sandirasegarane S, Smith N (2019). A pan-cancer perspective of matrix metalloproteases (MMP) gene expression profile and their diagnostic/prognostic potential. BMC Cancer.

[37] Abdel-Hamid NM, Abass SA (2021). Matrix metalloproteinase contribution in management of cancer proliferation, metastasis and drug targeting. Mol Biol Rep.

[38] Mondal S, Adhikari N, Banerjee S, Amin SA, Jha T (2020). Matrix metalloproteinase-9 (MMP-9) and its inhibitors in cancer: A minireview. Eur J Med Chem.

[39] Zeng Y, Gao M, Lin D, Du G, Cai Y (2022). Prognostic and Immunological Roles of MMP-9 in Pan-Cancer. Biomed Res Int.

[40] Chen L, Zhang J, He Y, Ding XY (2018). Matrix metalloproteinase-9 expression of GCTSC in peripheral tissue and central tissue of GCTB. J Cell Biochem.

[41] Huang H (2018). Matrix Metalloproteinase-9 (MMP-9) as a Cancer Biomarker and MMP-9 Biosensors: Recent Advances. Sensors (Basel).

[42] Roy R, Yang J, Moses MA (2009). Matrix metalloproteinases as novel biomarkers and potential therapeutic targets in human cancer. J Clin Oncol.

[43] Tian R, Li X, Gao Y, Li Y, Yang P, Wang K (2018). Identification and validation of the role of matrix metalloproteinase-1 in cervical cancer. Int J Oncol.

[44] Ma B, Ran R, Liao HY, Zhang HH (2021). The paradoxical role of matrix metalloproteinase-11 in cancer. Biomed Pharmacother.

[45] Lubowicka E, Zbucka-Kretowska M, Sidorkiewicz I, Zajkowska M, Gacuta E, Puchnarewicz A (2020). Diagnostic Power of Cytokine M-CSF, Metalloproteinase 2 (MMP-2) and Tissue Inhibitor-2 (TIMP-2) in Cervical Cancer Patients Based on ROC Analysis. Pathol Oncol Res.

[46] Chen DS, Mellman I (2017). Elements of cancer immunity and the cancer-immune set point. Nature.

[47] Turley SJ, Cremasco V, Astarita JL (2015). Immunological hallmarks of stromal cells in the tumour microenvironment. Nat Rev Immunol.

[48] Gajewski TF, Woo SR, Zha Y, Spaapen R, Zheng Y, Corrales L (2013). Cancer immunotherapy strategies based on overcoming barriers within the tumor microenvironment. Curr Opin Immunol.

[49] Gajewski TF (2015). The Next Hurdle in Cancer Immunotherapy: Overcoming the Non-T-Cell-Inflamed Tumor Microenvironment. Semin Oncol.

[50] Joyce JA, Fearon DT (2015). T cell exclusion, immune privilege, and the tumor microenvironment. Science.

[51] Salmon H, Franciszkiewicz K, Damotte D, Dieu-Nosjean MC, Validire P, Trautmann A (2012). Matrix architecture defines the preferential localization and migration of T cells into the stroma of human lung tumors. J Clin Invest.

[52] De Gregorio V, Urciuolo F, Netti PA, Imparato G (2020). In Vitro Organotypic Systems to Model Tumor Microenvironment in Human Papillomavirus (HPV)-Related Cancers. Cancers (Basel).

