# Supplementary material for: Integrated analysis of single-cell RNA-seq and bulk RNA-seq reveals MMP mediated expression patterns by distinct tumor microenvironment immune profiles in cervical cancer
Source: J Cancer. 2024 Aug 13;15(16):5258–76. doi: 10.7150/jca.96429 (PMC11375545; doi:10.7150/jca.96429)
Supplement: Supplementary file 1 — Supplementary figures and tables. [file jcav15p5258s1.pdf]

Supplementary material：

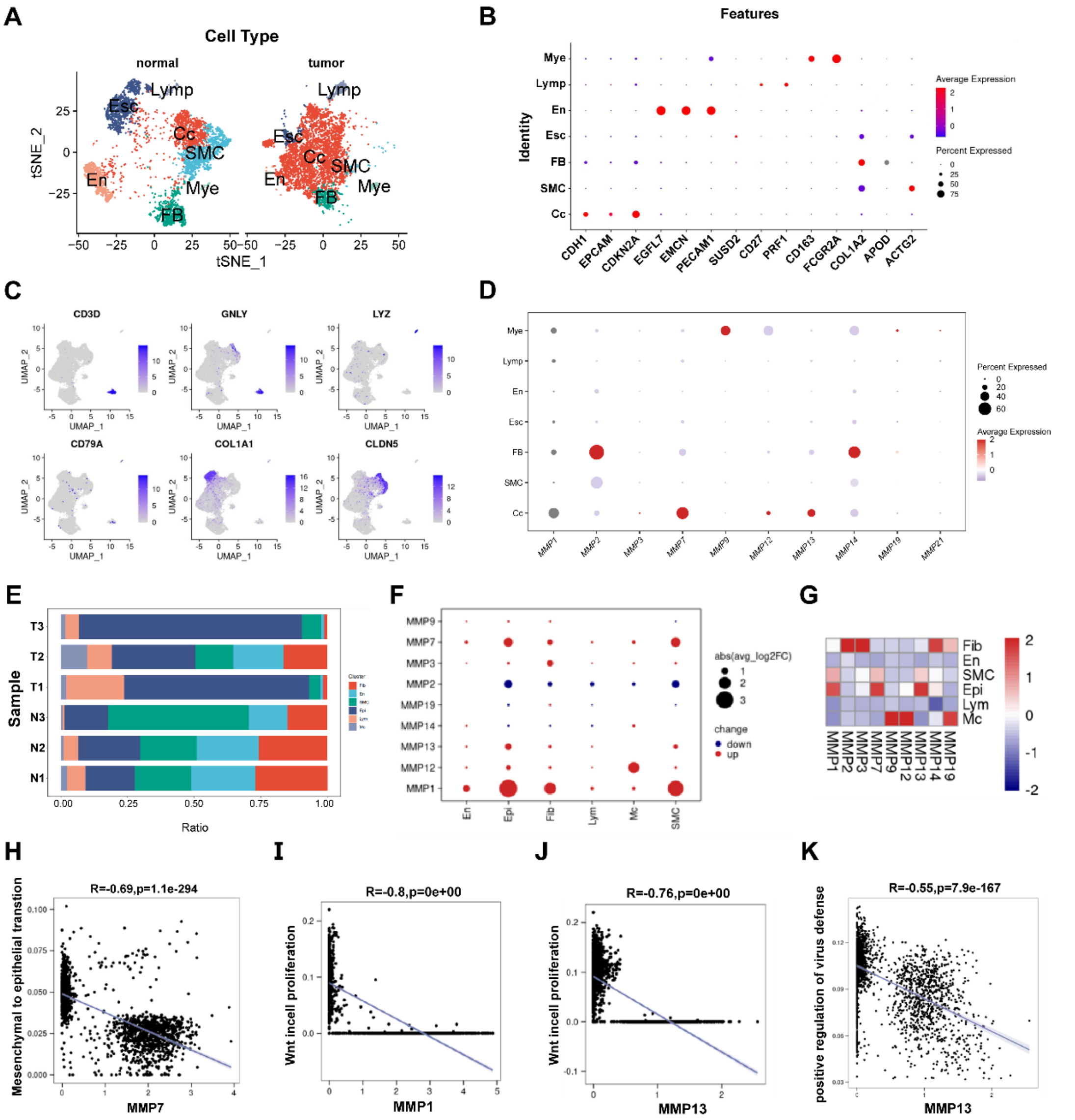

Figure S1 (A) Distribution and expression profiles of *MMP* across individual cell types in the normal and CC. tSNE of seven cell types. **(B)** This graph shows identified cell types and annotated using classical marker genes, the size of the dots indicates the average multiple of difference；And the color of the dots indicates high expression (red) or low expression (purple). **(C)** Expression levels of representative well-known markers across the cell types in CC. Color key from gray to purple indicates relative expression levels from low to high. **(D)** Differential expression of *MMPs* in different cell types of CC patients compared with control samples; The size of the dots indicates the average multiple of difference, and the color of the dots indicates up-regulated (red) or down-regulated (purple). **(E)** The proportion of each cell subtype after cell enrichment in BCL and CC samples. **(F)** Correlation scatter plots show the average gene expression of *MMPs* in all cell clusters. **(G)** Heatmap of *MMP* genes for six cell types. **(H-K)** Spearman correlation between *MMP-7* and EMT. Spearman correlation between *MMP-1* (*MMP-13*) and *Wnt* pathway in regulation of cell proliferation. Spearman correlation

between *MMP-13* and defense response to virus by host.

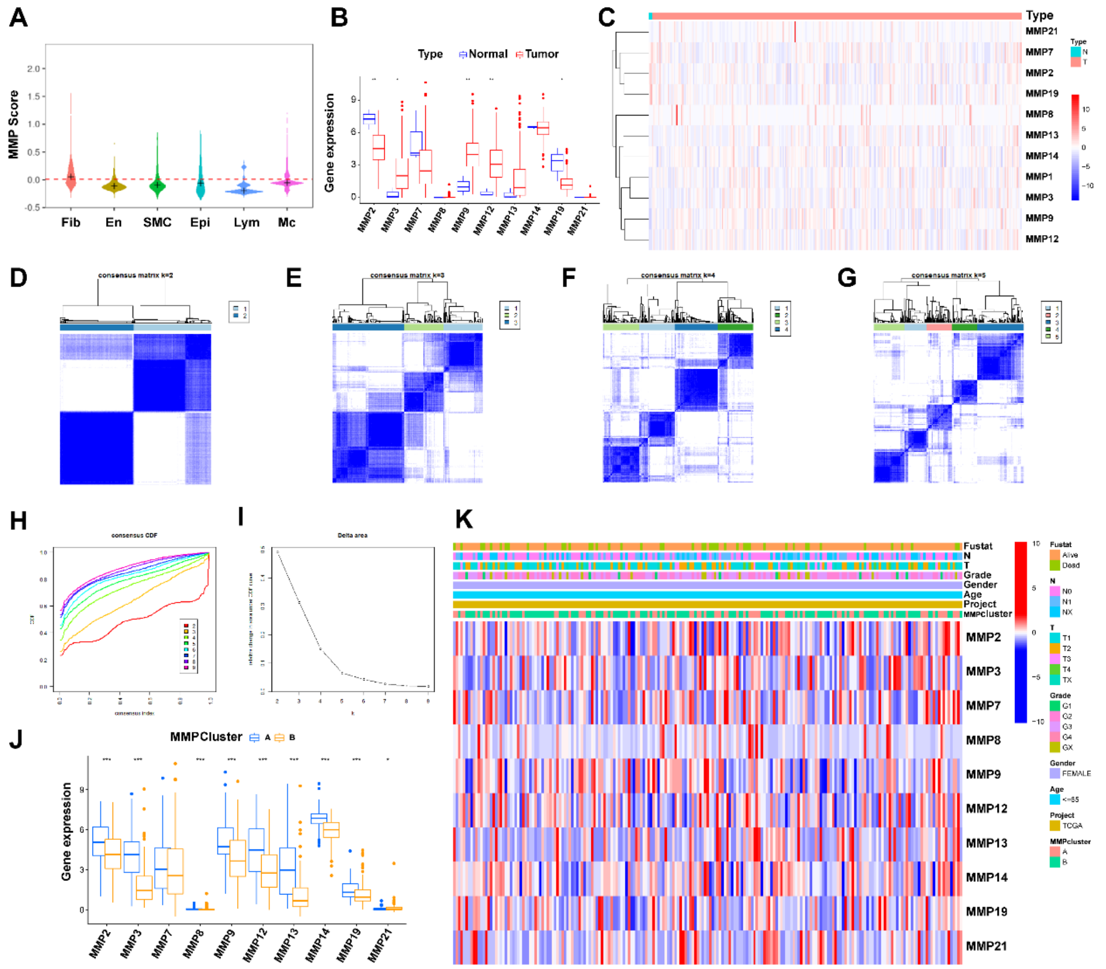

Figure S2 **(A)** MMPscore was ubiquitously expressed in all cell clusters. **(B)** The expression of 10 *MMPs* between normal patients and tumor patients. Tumor, red; Normal, blue. The upper and lower ends of the boxes represented interquartile range of values. The lines in the boxes represented median value, and dots showed outliers. **(C)** Expression heatmap of the 11 *MMPs* in normal and CC samples. Tumor, red; normal, blue. **(D)** Consensus matrices of the significant *MMPs* for k = 2. **(E-G)** Consensus matrices of the significant *MMPs* for k = (3-5). **(H-I)** Unsupervised clustering of *MMP* phenotype-related genes. Consensus CDF and Delta area were shown that k = 2 is the optimal solution. **(J)** The expression of 10 *MMPs* between blue MMPcluster-A and yellow MMPcluster-B. The upper and lower ends of the boxes meant interquartile range of values. The lines in the boxes represented median value, and dots showed outliers. The asterisks represented the statistical p value (* $p < 0.05$; ** $p < 0.01$; *** $p < 0.001$). **(K)** Unsupervised clustering of MMPclusters. The MMPcluster, node, tumor, grade, gender and age were used as patient annotations. Red represented high expression of regulators and blue represented low expression.

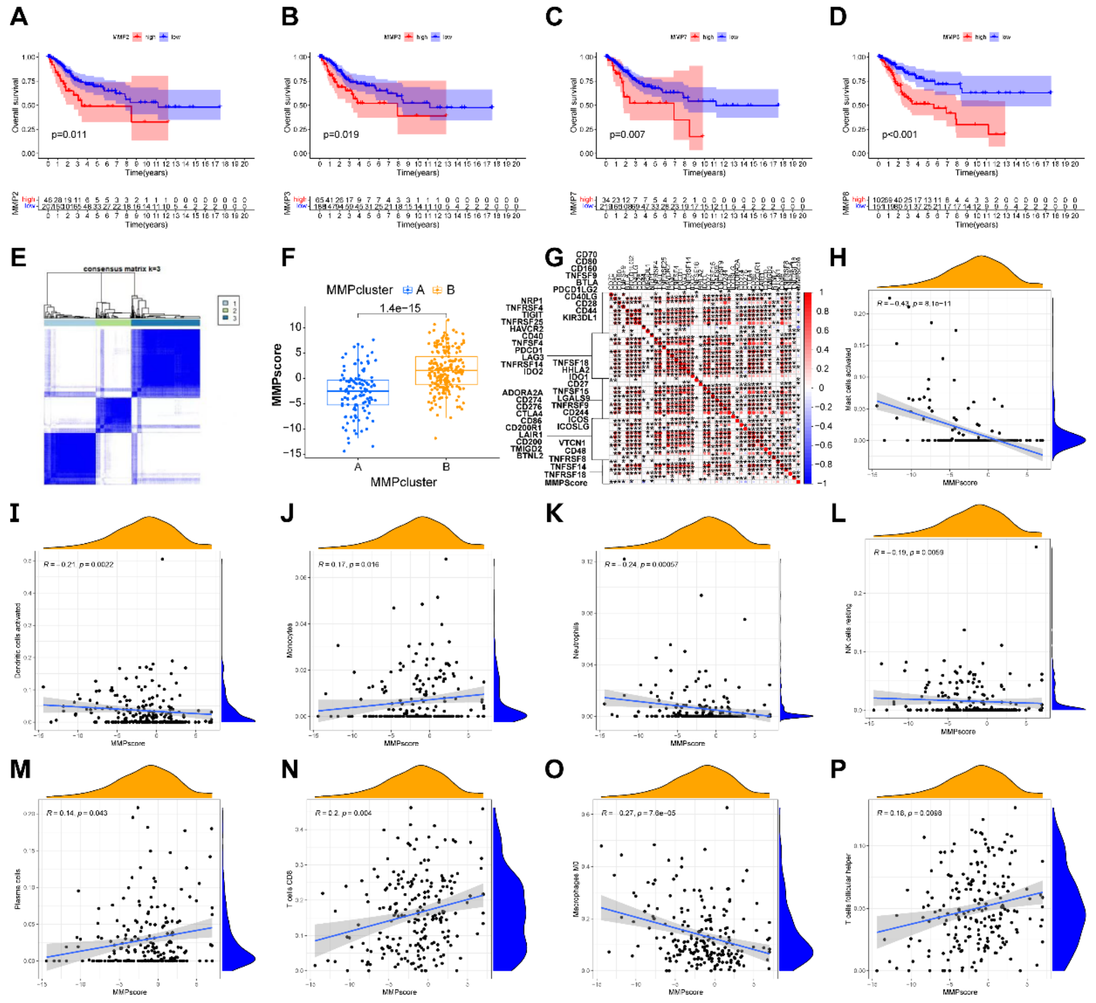

Figure S3 **(A)** Kaplan-Meier curves for high and low MMPscore patient groups in *MMP-2* cohort. Log-rank test, p = 0.011. **(B)** Kaplan-Meier curves for high and low MMPscore patient groups in *MMP-3* cohort. Log-rank test, p = 0.019. **(C)** Kaplan-Meier curves for high and low MMPscore patient groups in *MMP-7* cohort. Log-rank test, p = 0.007. **(D)** Kaplan-Meier curves for high and low MMPscore patient groups in *MMP-8* cohort. Log-rank test, $p < 0.001$. The asterisks represented the statistical p value (* $p < 0.05$; ** $p < 0.01$; *** $p < 0.001$). **(E)** Consensus matrices of the overlapping MMP phenotype-related genes for k = 3. **(F)** Differences in MMPscore among two MMPclusters. The Kruskal-Wallis test was used to compare the statistical difference between two MMPclusters ($p < 0.001$). **(G)** Correlation between MMPscore and immune checkpoints in CC. The color and the values meant the Spearman correlation coefficient. The asterisks indicated a statistically significant p -value calculated using Mann-Whitney U test (* $p < 0.05$; ** $p < 0.01$; *** $p < 0.001$). **(H)** Correlation between MMPscore and Mast cells activated in CC. $p < 0.001$. **(I)** Correlation between MMPscore and Dendritic cells activated in CC. p = 0.0022. **(J)** Correlation between MMPscore and Monocytes in CC. p = 0.016. **(K)** Correlation between MMPscore and Neutrophils in CC. p = 0.00057. **(L)** Correlation between MMPscore and NK cells resting in CC. p = 0.0059. **(M)** Correlation between MMPscore and Plasma cells in CC. p = 0.043. **(N)** Correlation between MMPscore and T cells CD8 in CC. p = 0.004. **(O)**

Correlation between MMPscore and Macrophages M0 in CC. $p < 0.001$. **(P)** Correlation between MMPscore and T cells follicular helper in CC. $p = 0.0098$.

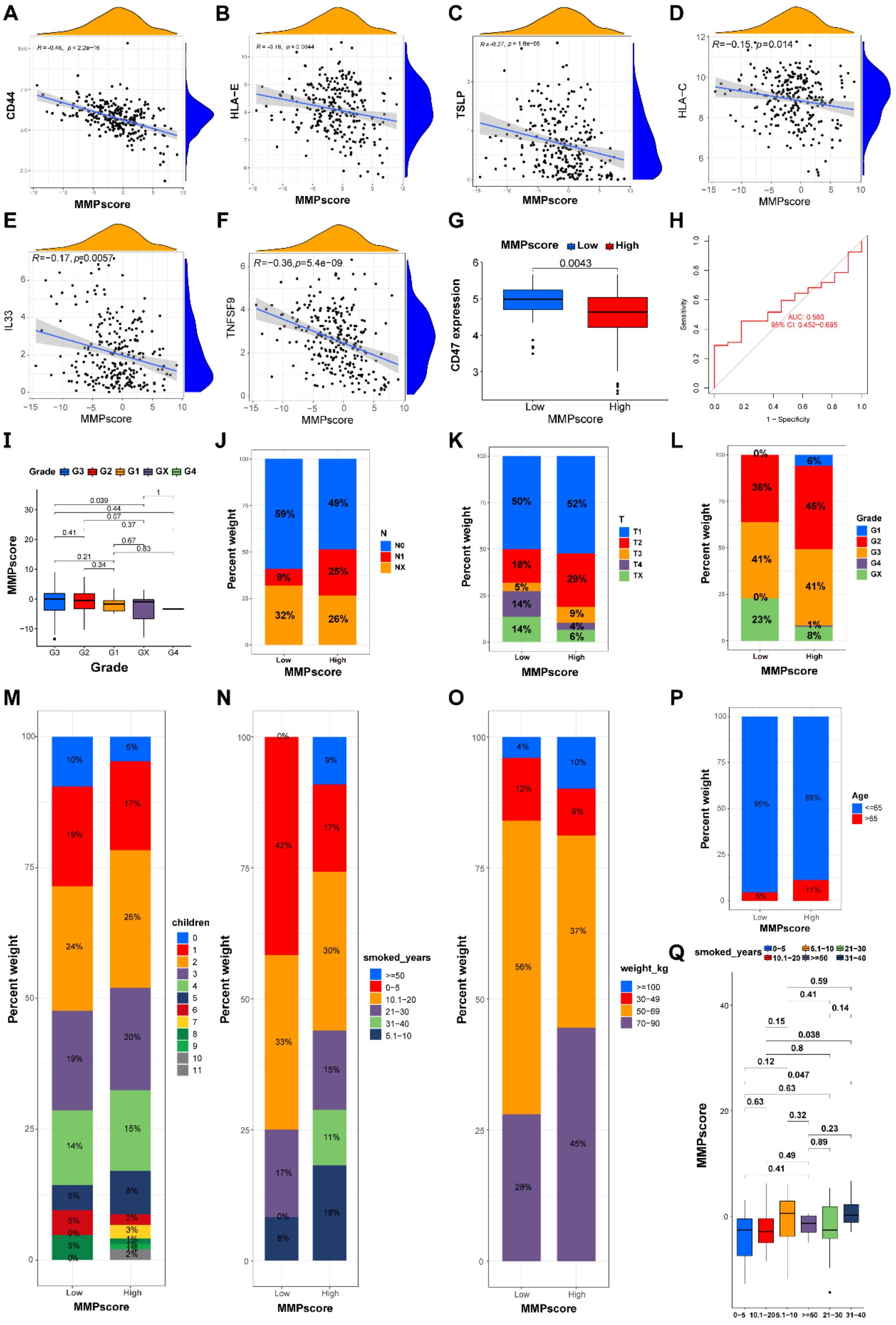

Figure S4 (A-C) Correlation between MMPscore and CD44, HLA-E, TSLP in CC. * p < 0.05, ** p < 0.01, and *** p < 0.001. **(D-F)** Correlation between MMPscore and

TNFSF9, IL33 and HLA-C in CC. * $p < 0.05$, ** $p < 0.01$, and *** $p < 0.001$. **(G)** Differences in CD47 expression among low MMPscore and high MMPscore clinical response groups. **(H)** Area under the ROC curve (AUC) of *MMP*. **(I)** Differences in MMPscore among distinct Grade clinical response groups. **(J)** The proportion of patients with N stage in the low or high MMPscore group. **(K)** The proportion of patients with T stage in the low or high MMPscore group. **(L)** The proportion of patients with Grade in the low or high MMPscore group. **(M)** The proportion of patients with children in the low or high MMPscore group. **(N)** The proportion of patients with smoked years in the low or high MMPscore group. **(O)** The proportion of patients with weight (kilogram) in the low or high MMPscore group. **(P)** The proportion of patients with age in the low or high MMPscore group. **(Q)** Differences in MMPscore among distinct smoke years clinical response groups.

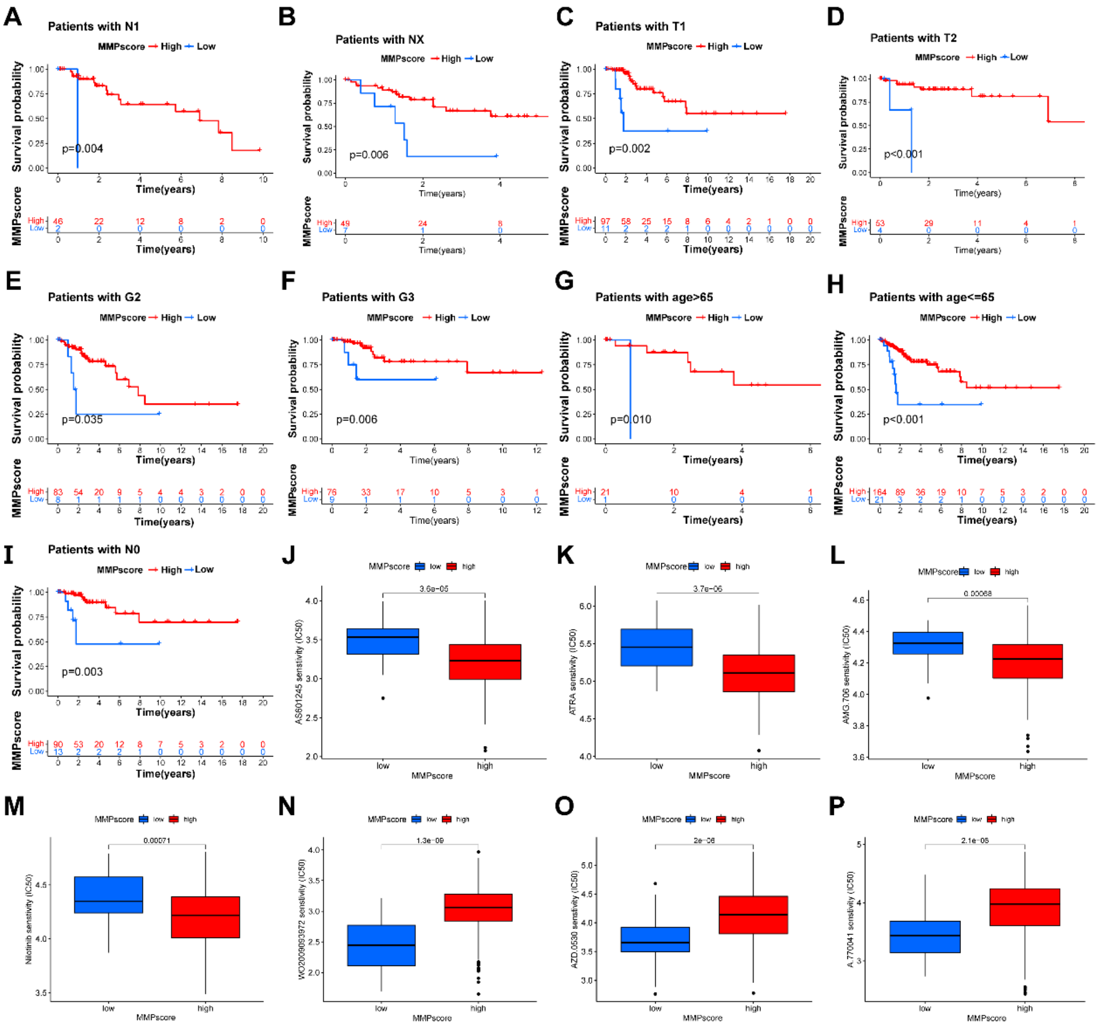

Figure S5 **(A)** Kaplan-Meier curves for high and low MMPscore patient groups in the patients with N1. Log-rank test, $p = 0.004$. **(B)** Kaplan-Meier curves for high and low MMPscore patient groups in the patients with NX. Log-rank test, p = 0.006. **(C)** Kaplan-Meier curves for high and low MMPscore patient groups in the patients with T1. Log-rank test, $p = 0.002$. **(D)** Kaplan-Meier curves for high and low MMPscore patient groups in the patients with T2. Log-rank test, p＜0.001. **(E)** Kaplan-Meier

curves for high and low MMPscore patient groups in the patients with G2. Log-rank test, $p = 0.035$. **(F)** Kaplan-Meier curves for high and low MMPscore patient groups in the patients with G3. Log-rank test, $p = 0.006$. **(G)** Kaplan-Meier curves for high and low MMPscore patient groups in the patients with age greater than 65. Log-rank test, $p = 0.010$. **(H)** Kaplan-Meier curves for high and low MMPscore patient groups in the patients with age less than 65. Log-rank test, $p < 0.001$. **(I)** Kaplan-Meier curves for high and low MMPscore patient groups in the patients with N0. Log-rank test, $p = 0.003$. **(J-P)** The correlation between the high and low expression of *MMP* and the semi-inhibited concentration sensitivity of various drugs. Drugs: AS601245, ATRA， AMG.706, Nilotinib, WO2009093972, AZD.0530, A.770041.

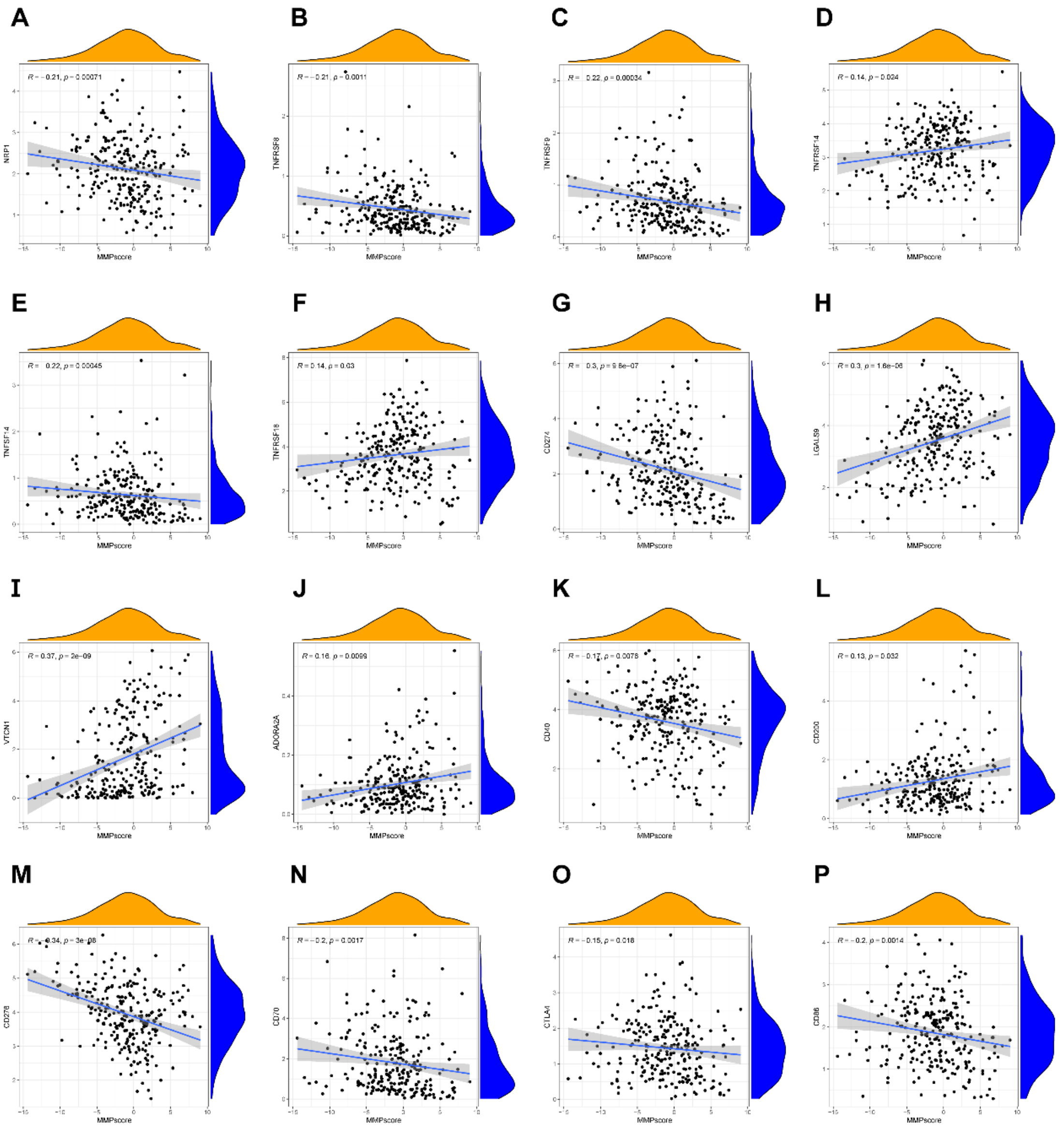

Figure S6 (A) Correlation between MMPscore and NRP1 in CC. * $p < 0.05$, ** $p < 0.01$, and *** $p < 0.001$. **(B)** Correlation between MMPscore and TNFRSF8 in CC. *

$p < 0.05$, ** $p < 0.01$, and *** $p < 0.001$. **(C)** Correlation between MMPscore and TNFRSF9 in CC. * $p < 0.05$, ** p < 0.01, and *** $p < 0.001$. **(D)** Correlation between MMPscore and TNFRSF14 in CC. * $p < 0.05$, ** $p < 0.01$, and *** $p < 0.001$. **(E)** Correlation between MMPscore and TNFSF14 in CC. * $p < 0.05$, ** $p < 0.01$, and *** $p < 0.001$. **(F)** Correlation between MMPscore and TNFRSF18 in CC. * $p < 0.05$, ** $p < 0.01$, and *** $p < 0.001$. **(G)** Correlation between MMPscore and CD274 in CC. * $p < 0.05$, ** $p < 0.01$, and *** $p < 0.001$. **(H)** Correlation between MMPscore and LGALS9 in CC. * $p < 0.05$, ** $p < 0.01$, and *** $p < 0.001$. **(I)** Correlation between MMPscore and VTCN1 in CC. * $p < 0.05$, ** $p < 0.01$, and *** $p < 0.001$. **(J)** Correlation between MMPscore and ADORA2A in CC. * $p < 0.05$, ** $p < 0.01$, and *** $p < 0.001$. **(K)** Correlation between MMPscore and CD40 in CC. * $p < 0.05$, ** $p < 0.01$, and *** $p < 0.001$. **(L)** Correlation between MMPscore and CD200 in CC. * $p < 0.05$, ** $p < 0.01$, and *** $p < 0.001$. **(M)** Correlation between MMPscore and CD276 in CC. * $p < 0.05$, ** $p < 0.01$, and *** $p < 0.001$. **(N)** Correlation between MMPscore and CD70 in CC. * $p < 0.05$, ** $p < 0.01$, and *** $p < 0.001$. **(O)** Correlation between MMPscore and CTLA4 in CC. * $p < 0.05$, ** $p < 0.01$, and *** $p< 0.001$. **(P)** Correlation between MMPscore and CD86 in CC. * $p < 0.05$, ** $p < 0.01$, and *** $p < 0.001$.

**Patient**		**Ca1**	**Ca2**	**Ca3**	**N1**	**N2**	**N3**
**Characteristic**	**Age**	41	62	72	51	53	43
	**Times of pregnancy**	5	1	2	3	3	2
	**Parity**	1	1	2	1	3	2
	**Tumor size (cm)**	2*1.5*1	6.2*4.4*6.33	3.5*3*1.8	-	-	-
	**Histology**	Adenocarcinoma	squamous	squamous	-	-	-
	**histological grading**	-	middle	low	-	-	-
	**Silva typing**	Silva C	-	-	-	-	-
	**HPV16/18**	HPV18(+)	HPV16(+)	HPV16(+)	-	-	-
	**FIGO staging**	IIIC1	III B	IB2	-	-	-
	**Lymph node metastasis**	positive	positive	negative	-	-	-
	**treatment**	surgery	chemotherapy	surgery			

Table S1 Comparison of basic data from eligible selected CC patients and controls. Including age, times of pregnancy, parity, tumor size, histology, histological grading or silva typing, HPV16/18, FIGO staging lymph node metastasis and treatment.

patient	Characteristic								
	Age	Times of pregnancy	Parity	Tumor size (cm)	Histology	histological grading	HPV16/18	FIGO staging	Lymph node metastasis
CC1	43	2	1	2.5*2*2	squamous	middle	16(+)	IB1	positive
CC2	35	4	3	2.5*1.6*0.9	squamous	middle	16(+)	IIIC1	positive
CC3	43	2	2	1.54*1.63*1	squamous	middle	16(+)	IIA1	negative
CC4	34	2	1	2.1*2.3*1.9	squamous	middle	16(+)	IB1	negative
CC5	42	2	2	2.8*2.5*1.5	squamous	middle-low	16(+)	IB2	negative
CC6	41	4	2	3.4*1.8*1.7	squamous	middle	18(+)	IIA1	negative
CC7	38	2	1	1*1*0.9	squamous	middle	16(+)	IB1	negative
CC8	44	4	2	2.5*2*1.5	squamous	middle	18(+)	IB2	negative
CC9	40	2	1	2.2*1.4*1	squamous	high-middle	16(+)	IB2	negative
CC10	38	3	2	2.1*2.1*3.4	squamous	middle	16(+)	IB2	negative
CC11	46	4	3	5.5*3.6*1.3	squamous	middle	16(+)	IIICIP	negative
CC12	34	3	1	1.4*1.6*2.9	squamous	middle	16(+)	IIA2	negative
N1	47	6	3	-	-	-	-	-	-
N2	43	3	1	-	-	-	-	-	-
N3	45	2	2	-	-	-	-	-	-
N4	47	1	1	-	-	-	-	-	-
N5	34	1	1	-	-	-	-	-	-
N6	44	3	1	-	-	-	-	-	-
N7	47	3	1	-	-	-	-	-	-
N8	49	3	2	-	-	-	-	-	-
N9	47	1	1	-	-	-	-	-	-
N10	48	4	1	-	-	-	-	-	-
N11	48	1	1	-	-	-	-	-	-
N12	44	2	1	-	-	-	-	-	-

Table S2 Comparison of basic data from eligible selected CC patients and controls. Pathological paraffin blocks of patients were used for immunohistochemical experiments. Including age, times of pregnancy, parity, tumor size, histology, histological grading, HPV16/18, FIGO staging and lymph node metastasis.
